# RNA-tethering assay and eIF4G:eIF4A obligate dimer design uncovers multiple eIF4F functional complexes

**DOI:** 10.1093/nar/gkaa646

**Published:** 2020-08-04

**Authors:** Francis Robert, Regina Cencic, Renying Cai, T Martin Schmeing, Jerry Pelletier

**Affiliations:** Department of Biochemistry, McGill University, Montreal, Canada; Department of Biochemistry, McGill University, Montreal, Canada; Department of Biochemistry, McGill University, Montreal, Canada; Department of Biochemistry, McGill University, Montreal, Canada; Department of Biochemistry, McGill University, Montreal, Canada; Department of Oncology; Rosalind & Morris Goodman Cancer Research Centre, McGill University, Montreal, Canada

## Abstract

Eukaryotic cellular mRNAs possess a 5′ cap structure (m^7^GpppN) which plays a critical role in translation initiation mediated by eukaryotic initiation factor (eIF) 4F. The heterotrimeric eIF4F complex possesses several activities imparted by its subunits that include cap recognition (by eIF4E), RNA unwinding (eIF4A), and factor/ribosome recruitment (eIF4G). Mammalian cells have paralogs of all three eIF4F subunits and it remains an open question as to whether these all can participate in the process of ribosome recruitment. To query the activities of the eIF4F subunits in translation initiation, we adopted an RNA-tethering assay in which select subunits are recruited to a specific address on a reporter mRNA template. We find that all eIF4F subunits can participate in the initiation process. Based on eIF4G:eIF4A structural information, we also designed obligate dimer pairs to probe the activity of all combinations of eIF4G and eIF4A paralogs. We demonstrate that both eIF4GI and eIF4GII can associate with either eIF4A1 or eIF4A2 to recruit ribosomes to mRNA templates. In combination with eIF4E and eIF4E3, our results indicate the presence of up to eight eIF4F complexes that can operate in translation initiation.

## INTRODUCTION

Cellular translational flux is largely determined by the rate-limiting phase of protein synthesis—translation initiation (1,2). Eukaryotic initiation factor (eIF) 4E—a protein critical for the recruitment of ribosomes to capped cellular mRNAs is the least abundant translation factor in HeLa cells (3,4). In eukaryotes, ribosome recruitment is governed by the eIF4F complex which consists of the eIF4E cap binding protein, the eIF4A DEAD-box RNA helicase, and the eIF4G scaffolding protein. Interactions between eIF4G and ribosome-bound eIF3, stimulated and stabilized by the RNA binding protein eIF4B, underlie the recruitment of ribosomes to mRNA templates (5,6). Our understanding of the mechanism of eIF4F-dependent ribosome recruitment to mRNAs is still rudimentary (7).

**Figure 1.**
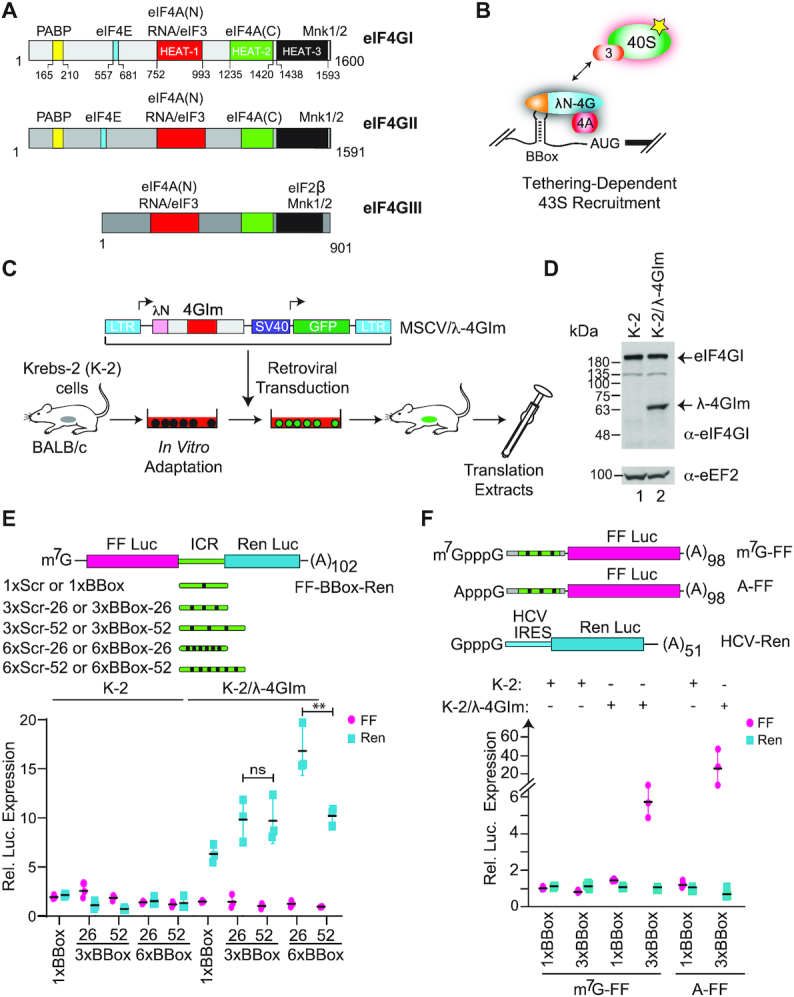
(**A**) Schematic representation of eIF4G homologs highlighting functional domains. The numbering is based on Gene Accession NM_182917.4 (eIF4GI), NM_001198802.2 (eIF4GII) and NM_001418.4 (eIF4GIII). Domain organization is based on Marintchev *et al.* (49). (**B**) Schematic diagram illustrating internal recruitment of 40S ribosomes and associated initiation factors (represented by a yellow star) via a λN-4G fusion, based on the tethering assay (21). (**C**) Schematic representation of retroviral-based expression system for ectopic production of eIF4GI in cells. Shown is the vector designed to express the middle domain of eIF4GI (amino acids 653–1131) fused to two λN domains. (**D**) Western blot denoting expression of λ–4GIm in translation extracts prepared from MSCV/λ–4GIm—infected K-2 cells (K-2/λ–4GIm). (**E**). **Top:** Schematic representation of bicistronic reporters used in this study. Shown are variations in the inter-cistronic region (ICR) tested. BBoxes or scrambled sequence controls (Scr) are present one (1×), three (3×) or six (6×) times, with a spacing of 26 or 52 nucleotides. **Bottom:** Luciferase production following *in vitro* translation of 10 μg/ml of the indicated mRNA reporters in either K-2 or K-2/λ-4GIm extracts. Values are normalized to the Scr control mRNAs of the same length and copy number (see Supplementary Figure S2A) and represent the average of 5 biological replicates, with each experiment performed in technical duplicates. ± SD. ns, not significant, **P = 0.02. (**F**). **Top:** Schematic representation of monocistronic reporters used in this study. **Bottom:** Luciferase production following *in vitro* translation of 4 μg/ml of the indicated mRNA reporters in the indicated extracts. Values are normalized to the Scr control mRNAs of the same length. *n* = 5 biological replicates, ± SD.

Mammalian cells encode several eIF4E, eIF4A and eIF4G paralogs. Human eIF4GI and eIF4GII (confusingly also referred to as eIF4G3) are 48% identical, multi-domain proteins that interact with eIF4E, eIF4A, RNA, poly (A) binding protein (PABP), eIF3, and the Mnk1 and Mnk2 kinases (Figure 1a). The eIF4G paralogs likely differentially participate in translation initiation as they appear to be regulated by different kinases and display disparate sensitivities to viral (e.g. poliovirus, HIV, retroviral) proteases and caspase-3 during apoptosis (8). In addition to their central role in cap-dependent translation, eIF4GI and eIF4GII are also required for initiation on some IRESes. eIF4G can be recruited to IRESes via IRES-transacting factors (ITAFs), thus bypassing the requirement for a 5′ cap structure while maintaining the ability to harness eIF4A helicase activity (7). Following infection by some picornaviruses or retroviruses, or in response to caspase-3 activation, cap-dependent translation is compromised due to cleavage of eIF4G between the NTD-located eIF4E binding site and the RNA/eIF4A binding sites—an event that favors IRES-mediated translation (7). Cells also synthesize a third eIF4G homolog, eIF4GIII (also known as p97, Dap5, Nat1), that lacks the eIF4E binding site and that has been implicated in internal initiation of cellular translation (Figure 1A). eIF4GIII is required for the translation of specific proteins required for embryonic stem cell differentiation (9).

Human eIF4A1 and eIF4A2 share 90% amino acid identity, both can interchange into the eIF4F complex (10), and in HeLa cells, eIF4A1 is the more abundant homolog (4). The two proteins are differentially expressed depending on cell growth status – proliferating cells express more eIF4A1 mRNA whereas at growth arrest, eIF4A2 mRNA levels increase (3-fold) and eIF4A1 mRNA levels diminish (∼10–20%) (11). As well, transcription of eIF4A1 is under MYC regulation, whereas that of eIF4A2 is not (12). eIF4A2 has also been identified as a host factor required for efficient HIV-1 replication (13). As well, eIF4A1 but not eIF4A2, is a target for cleavage by foot-and-mouth disease virus 3C protease during viral infection (14). These examples of distinct regulation and translational requirements allude to potential different roles in translation. Paradoxically, a recent report has suggested that eIF4A2 is a suppressor of translation and mediates repression by microRNAs (15). A third eIF4A paralog, eIF4A3 is predominantly nuclear, a core component of the exon junction complex and involved in nonsense-mediated decay (16).

There are three eIF4E paralogs, eIF4E (aka eIF4E1), eIF4E2 (aka 4EHP) and eIF4E3 but only two can bind to the N-terminal domain (NTD) of eIF4G: eIF4E and the lesser studied protein, eIF4E3 (17). There are a limited number of studies examining the biochemical properties of eIF4E3. It binds the cap in an atypical manner in that it does not use two aromatic amino acids for cation-π stacking with the m^7^G moiety, but rather only has one aromatic amino acid that can participate in π-stacking (18). This is likely responsible for the 10–40-fold lower affinity of eIF4E3 for cap analogs, compared to eIF4E (18). Overexpression of eIF4E3 followed by analysis of gene expression changes across polysomes has revealed that elevated levels of eIF4E3 can alter the translatome, however, it remains to be determined if this was a direct or secondary consequence of the long-term overexpression conditions used (19). Hence, whether eIF4E3 can directly participate in the ribosome recruitment process is an open question. In sum, these studies point to the possible existence of eight cellular eIF4F isoforms: four containing eIF4E and four with eIF4E3, although experiments delineating their activities have not been reported. Neither is it known if all combinations of eIF4G and eIF4A homologs have ribosome recruitment activity.

In RNA biology, tethering assays have proven to be powerful approaches by which RNA binding constraints are removed from proteins to enable functional characterization of effector domains. A very useful system is the λ phage anti-terminator protein, N, which can be fused to proteins of interest to monitor their effects on mRNA reporters harboring specific λN binding sites (BBoxes) (20). Such assays (Figure 1B) have been previously applied to study the roles of eIF4E, eIF4G and eIF4A in translation initiation (21,22). Specifically, the Hentze lab has shown that the middle domain of eIF4GI is sufficient for ribosome recruitment. They also demonstrated that internal recruitment of λ-eIF4E to an mRNA template harboring BBoxes was capable of recruiting ribosomes and that this was likely via its assembly into the eIF4F complex (21,22). Herein, we revisit the tethering assay to: (i) probe the roles of the different eIF4E, eIF4A and eIF4G paralogs in translation, (ii) develop eIF4G:eIF4A obligate dimer pairs to demonstrate that all four combinations of eIF4GI, eIF4GII, eIF4A1 and eIF4A2 participate in the ribosome recruitment process and (iii) probe the requirements for eIF4A1 activity in ribosome recruitment. Our results define the potential existence of eight mammalian eIF4F functional complexes and provide obligate dimers of eIF4G:eIF4A that will be useful in delineating individual subunit activity.

## MATERIALS AND METHODS

### Expression vectors

Expression constructs were generated by cloning of G blocks, PCR fragments, or gene synthesis fragments into MSCV-IRES-GFP. FLuc and RLuc reporters are pKSII+ based and have been previously described (23). Following cloning, all vector products were sequence verified. Expression vectors encoding eIF4A paralogs are N-terminally tagged with a 3× HA tag whereas all other expression vectors have a 1× FLAG tag. All plasmids, vector maps, and sequences are available upon request.

### Hippuristanol

Synthesis of hippuristanol (Hipp) used in this study has been previously described (24).

### Krebs-2 cells

Krebs-2 (K-2) ascites tumor cells were passaged in the peritoneal cavity of female BALB/c mice. Frozen stocks were made by diluting ascites fluid with an equal volume of PBS containing 20% DMSO. To adapt these cells to grow in culture, they were maintained in B-cell Media (BCM; 45% DMEM, 45% IMDM, 55 μM ß-mercaptoethanol, 10% FBS and Glut/Pen/Strep) on γ-irradiated *Arf^−^^/^^−^* MEF feeder layers. K-2 cells were split 1:3 every 2–3 days. To generate K-2/λ-4GIm extracts, cells were transduced with MSCV-λ-4GIm retrovirus generated following retroviral packaging using ecotropic Phoenix cells according to established protocols (http://www.stanford.edu/group/nolan/retroviral_systems/retsys.html). Transduced cells were sorted on a FACSAria II (BD Biosciences) to obtain a 100% GFP^+^ cell population. GFP^+^ cells were passaged through mice twice before preparing translation competent extracts as described previously (25), except that ascites cells were not incubated *in vitro* for 2 h at 37°C before extract preparation. *In vitro* translations were performed as described previously (26).

### Assessment of *in cellula* expression activity

HEK293T cells were maintained at 37°C in DMEM supplemented with 10% FBS and Glut/Pen/Strep. Prior to transfection, 10^6^ cells were seeded in each well of a six-well plate. The following day, cells were transfected with 3 μg of each construct using PEI (27). The next morning transfected cells were trypsinized, resuspended in 3 ml of DMEM, and 400 μl of cells were re-plated into four wells of a 24-well plate. Six hours later, 200 ng of either the 3xBBox-FF or 3xScr-FF mRNA reporters, along with 50 ng of HCV-Ren mRNA was transfected in 200 μl of OptiMEM using 1 μl of DMRIE-C reagent according to the manufacturer's recommendations (Thermo Fisher Scientific). Cells were lysed 12–16 h later using Passive Lysis Buffer (Promega) and FF and Ren luciferase activity determined on a Fluostar 96-well plate reader BMG Labtech (28). For obligate dimer testing, 2 μg of each construct were co-transfected. To make up differences in plasmid amounts when single constructs were transfected, MSCV/λ-SVgfp empty vector was used—thus ensuring all transfections had the same final DNA concentration. For Hipp treatment, 150 nM of compound was added 30 min following mRNA transfections.

### Expression and immunoprecipitation assays

The expression of each construct was assessed by Western blotting. To this end, cells were transfected with 3 μg of DNA as described above. The next morning, media was refreshed, and cells were cultured at 37°C for an additional 24 h. Cells were then washed with PBS, lysed in RIPA buffer (20 mM Tris–HCl [pH 7.6], 100 mM NaCl, 1 mM EDTA, 1 mM EGTA, 1% NP40, 0.5% sodium deoxycholate, 0.1% SDS, 10 mM NaF, 20 mM β-glycerophosphate, 1 mM PMSF, 4 μg/ml aprotinin, 2 μg/ml leupeptin, 2 μg/ml pepstatin, 1 mM DTT), resolved on a 10% SDS-PAGE, and transferred to PVDF membranes (Bio-Rad). Antibodies used in this study were the following: α-eEF2 (Cell Signaling, 2332), α-GAPDH (Abcam, 8245), α-FLAG (M2 Sigma, F1804), α-HA (Cell Signaling, 2367), α-eIF4A (Santa Cruz Biotechnology, sc-50354), α-GCN4 (Absolute Antibodies, AB00436-1.1), α-hNRNPA1 (Cell Signaling, 4296), α-eIF4GI (Bethyl, A300-502A), and α-eIF4G (Cell Signaling, 2498). Anti-GAPDH or α-eEF2 antibodies were used interchangeably as loading controls for Western blots.

Immunoprecipitations were performed by seeding 10 million HEK293T cells in 10 cm dishes. The next day, cells were transfected with 15 μg of control vector or 7.5 μg of each obligate dimer mutant pair as described above and medium was refreshed 24 h later. Forty-eight hours following transfections, cells were washed with PBS and collected. Cells were centrifuged for 10 min at 4°C at 300 × g and the pellet was lysed with 400 μl of NP40 lysis buffer (20 mM Tris–HCl [pH7.5], 150 mM NaCl, 0.5% NP40, 2 mM EDTA, 10 mM NaF, 20 mM β-glycerophosphate, 1 mM PMSF, 4 μg/ml aprotinin, 2 μg/ml leupeptin, 2 μg/ml pepstatin, 1 mM DTT) and incubated on ice for 10 min. The lysate was then cleared by centrifugation at 10 000 × g for 10 min at 4°C, the supernatant was transferred into a new tube and protein content quantitated using the DC protein assay (Biorad). Protein (750 μg) in 400 μl of lysate was prepared and 40 μl kept as input for Western blot analyses. The remaining lysate was added to washed anti-FLAG-M2 magnetic beads to immunoprecipitate eIF4G paralogs. To prepare the beads, 20 μl of anti-FLAG-M2 magnetic beads (Millipopre-Sigma M8823) were washed twice with NP40 lysis buffer and recovered using a magnet. Beads and lysates were incubated end-over-end at 4°C overnight and beads collected by centrifugation. The beads were then washed end-over-end five times for 10 min at 4°C with 500 μl of lysis buffer. After the last wash, the beads were resuspended in 40 μl of 1× SDS loading buffer and 20 μl was used for Western blotting. In experiments involving obligate dimers, α-FLAG and α-HA antibodies were used to detect eIF4G and eIF4A, respectively.

### RT-qPCR

HEK293T cells were transfected as described above and RNA isolated 48 h later using TRIzol according to the manufacturer's recommendations (Thermo Fisher Scientific). One microgram of RNA was used in a 20 μl reverse transcriptase reactions containing M-MuLV-RT and oligo d(T)_23_VN as primer following the protocol provided by the manufacturer (New England BioLabs). One microliter of a 10-fold dilution of the reverse-transcriptase reaction was used in qPCRs on a CFX96 PCR machine (BioRad) with FLuc-specific oligonucleotides (Fwd: ^5′^TCGAAATGTCCGTTCGGTTG^3′^; Rev: ^5′^TACGGTAGGCTGCGAAATGTT^3′^) or GAPDH-specific oligonucleotides (Fwd: ^5′^GGTATCGTGGAAGGACTCAT^3′^; Rev: ^5′^GCAGGGATGATGTTCTGGAG^3′^) and SsoFast EvaGreen Supermix (BioRad).

### Quantification and statistical analysis

Statistical analyses were performed using SigmaPlot 11. Unless otherwise stated, ‘*n* = 3’ refers to three biological replicates each performed in technical duplicates.

## RESULTS

### *In vitro* tethered-based, ribosome recruitment assay

To establish the parameters of a tethering assay to measure ribosome recruitment, we fused the λN RNA binding domain to the middle fragment of eIF4GI (Figure 1E, 4GIm) previously shown to be sufficient to mediate ribosome recruitment (21). This domain harbors ten alpha helices arranged into five HEAT repeats and is sufficient for eIF4A1 binding, RNA interaction, and 48S ribosome recruitment (29).

Krebs-2 (K-2) cells have been continuously passaged *in vivo* since they were first adapted to grow as an ascites tumor in Balb/c mice in 1951 (30). These cells have been extensively used to generate high-quality, cap-dependent translation extracts (25). We successfully adapted these cells to *in vitro* culturing conditions, enabling us to retrovirally transduce them, select GFP^+^ infected cells, passage the infected tumor cells in mice, and prepare *in vitro* translation extracts expressing λ-4GIm (Figure 1C, D). We then tested the ability of these extracts to stimulate translation from reporters harboring BBoxes, either positioned within the intercistronic region of a bicistronic reporter (Figure 1E, top panel) or placed within the 5′ leader of a monocistronic reporter (Figure 1F, top panel). In the bicistronic context, we tested the ability of reporters harboring 1×, 3× or 6× BBoxes to stimulate translation of the downstream renilla open reading frame (ORF). We also assessed the consequence of separating the BBoxes by 26 or 52 nucleotides. Control reporters harbored scrambled (Scr) sequences instead of BBoxes (Supplementary Figure S1). The Scr-containing reporters were unable to serve as templates for translation initiation and behaved similarly when used to program translation extracts prepared from K-2 cells (Supplementary Figure S2A). The 1×BBox, 3×BBox and 6×BBox-containing reporters also showed little activity when used to program K-2 cell extracts (Figure 1E). However, significant Ren expression was observed when BBox-containing mRNAs were used to program λ-FLAG-4GIm containing K-2 extracts (K-2/λ-4GIm) (Figure 1E). Reporters with 3× and 6×BBox were stimulated more than the 1×BBox mRNA. With the 3×BBox mRNA, this stimulation was ∼10-fold (relative to 3×Scr controls) and did not change if the distances between the BBoxes were 26 or 52 nucleotides. Better stimulatory activity (∼15-fold; relative to 3×Scr controls) was observed with 6×BBoxes spaced 26 nucleotides apart, although when the distance was increased to 52 nucleotides, activity at par with the 3×BBox mRNA reporter was observed.

The effects of BBox spacing and copy number variation was also examined in the monocistronic context (Figure 1F, Supplementary Figure S2B). As noted in the bicistronic context, luciferase output did not scale when comparing 3× versus 6×BBox mRNA reporters, but were better than obtained with 1×BBox mRNA (Figure 1F, Supplementary Figure S2B). The monocistronic context also allowed us to compare the behavior of m^7^G- versus A-capped mRNAs (Figure 1F). In K-2/λ-4GIm extracts, we observed higher levels of λ-FLAG-4GIm-dependent translation stimulation of FF luciferase (∼4-fold) from A-capped versus m^7^G-capped mRNAs (Figure 1F). We tentatively attribute this difference in activity to the absence of competition between eIF4F- and λ-4GIm-mediated initiation on A-capped mRNA templates. This thus provides a sensitive, BBox-dependent readout for monitoring translation initiation.

### *In cellula* tethered-based ribosome recruiting assay

**Figure 2.**
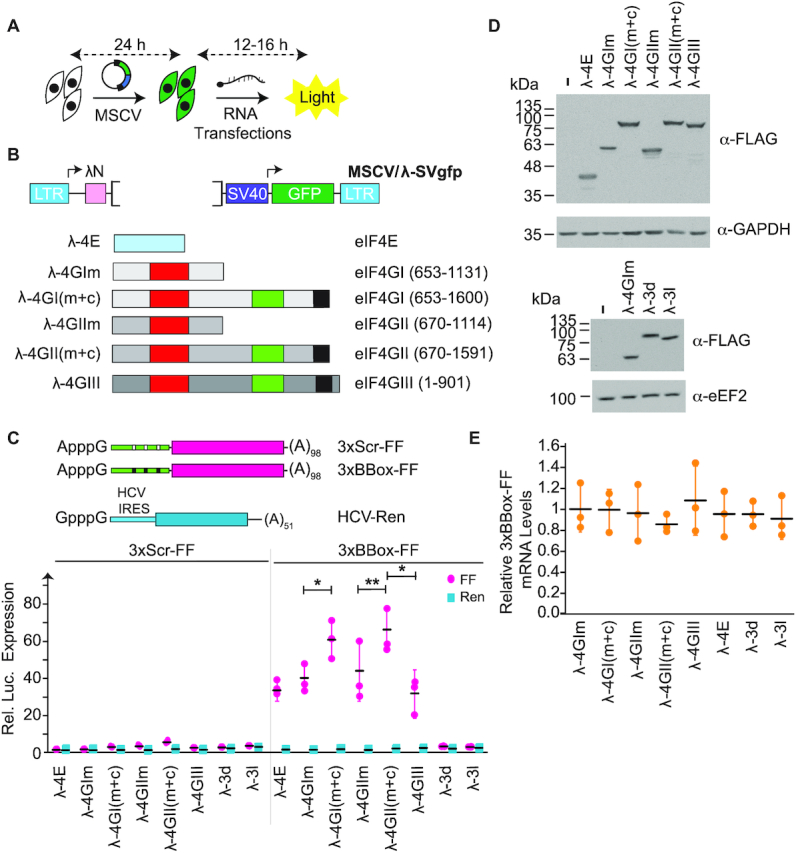
(**A**) Schematic diagram illustrating the order of transfection events in the *in cellula* tethering assay. Cells are first transfected with expression vectors driving synthesis of λ-fusions and GFP. One day later, mRNA reporters are transfected and luciferase values determined 12–16 h later. (**B**) Schematic representation of eIF4E and eIF4G expression constructs. Numbers in parenthesis denote amino acid positions for the eIF4G homologs. Functional domains defined in Figure 1A are shown for reference. (**C**) Luciferase production following co-expression of the indicated monocistronic mRNAs and MSCV expression vectors in HEK293T cells. Values are normalized to cells having received empty MSCV expression vector. *n* = 3, ± SD. *P < 0.01; **, 0.05> *P* >0.01. (**D**) Western blot documenting expression levels of λ-fusions in transfected HEK293T cells. (**E**) Assessment of A-capped 3×BBox-FF mRNA levels in transfected cells by RT-qPCR. Values are expressed relative to cells having received empty MSCV/λ-SVgfp expression vector. *n* = 3, ±SD.

We then sought to test the response of the A-capped, 3×BBox mRNA reporter to a series of λ-fusions *in cellula* (Figure 2A). Here, we assessed eIF4E, the middle (m) and two-thirds C-terminal (m+c) domains of eIF4GI and eIF4GII, as well as full-length eIF4GIII (all with N-terminal FLAG tags), for their ability to stimulate translation in this setting (Figure 2B). We also queried the activity of two eIF3 subunits, eIF3d and eIF3l, which have been reported to harbor m^7^G cap-binding activity to see if these were sufficient for recruiting ribosomes (31,32). We found that eIF4E and all eIF4G fusions were capable of stimulating translation in a BBox-dependent manner, whereas eIF3d and eIF3l showed no activity in our assay (Figure 2C). The eIF4G(m+c) domains were slightly more active (∼30%) than the eIF4Gm domains or full length eIF4GIII. All recombinant proteins were expressed to similar levels (Figure 2D) and no significant differences in levels of 3×BBox and 3×Scr FF mRNAs were noted (Figure 2E). eIF3d and eIF3l were unable to stimulate translation in this assay (Figure 2C). These experiments demonstrate that all three eIF4G paralogs are capable of mediating robust stimulation of translation in this BBox-dependent system.

To define the minimum translation stimulation domain (mTSD) of eIF4GIm, we generated a series of deletion mutants (Supplementary Figure S3A). Deletion of the HEAT domains within the HEAT/MIF4G region (29) produced a protein no longer capable of stimulating translation (4GIm(Δ816–953)) (Supplementary Figure S3B). C-terminal deletions of up to 78 aa (4GIm(653–1053)) retained wild type (wt) activity, but beyond this (4GIm(653–1020)) failed to stimulate translation. A modest reduction in activity (∼30%) was observed with an N-terminal deletion that removed the first 33 aa of eIF4GIm (686–1131) and this effect was exacerbated with truncations extending beyond this region (Supplementary Figure S3B). All constructs expressed similar levels of proteins (Supplementary Figure S3C). These studies define the eIF4GIm mTSD as spanning amino acids 653–1053.

**Figure 3.**
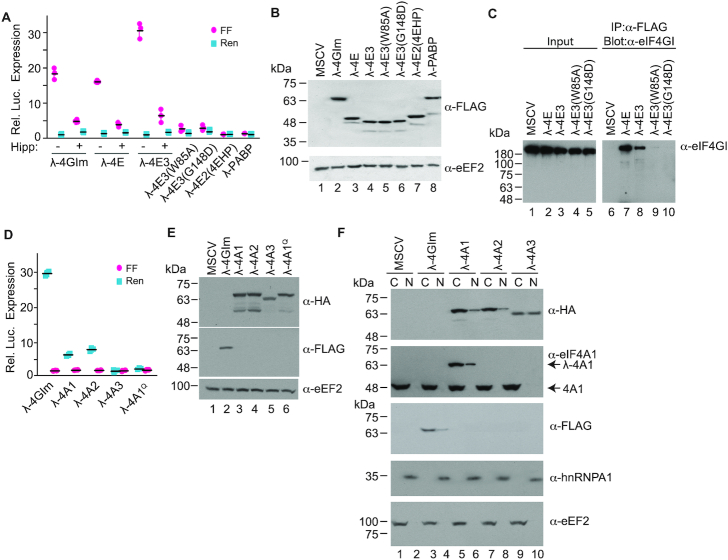
(**A**) Luciferase production following co-expression of the indicated MSCV expression vectors in HEK293T cells with 3×BBox mRNA. *n* = 3, ±SD. (**B**) Western blot documenting expression levels of λN fusions in transfected HEK293T cells. (**C**) Immunoprecipitations performed from cell extracts expressing the indicated FLAG-tagged λN-4E fusion proteins. Following SDS-PAGE, Western blots were performed with antibodies targeting eIF4GI. (**D**) Luciferase production following co-expression of the indicated MSCV-eIF4A expression vectors in HEK293T cells with 3×BBox mRNA. *n* = 3, ±SD. (**E**) Western blot documenting expression levels of λN fusions in transfected HEK293T cells. (**F**) Western blot documenting subcellular localization (C, cytoplasmic; N, nuclear) of the indicated λN fusions in transfected HEK293T cells. eEF2 and hnRNPA1 are used as loading control for cytoplasmic and nuclear fractionation, respectively.

Given the ability of eIF4E to stimulate translation in the tethering assay (Figure 2C and (22)), we also examined this property for two other FLAG-tagged cap-binding proteins, 4EHP and eIF4E3 (33). 4EHP is a repressor of translation and does not interact with eIF4G, whereas eIF4E3 can interface with eIF4G (33). Like λ-4GIm and λ-4E, λ-4E3 was able to stimulate translation from the 3xBBox mRNA reporter (Figure 3A). To probe whether eIF4A was required for ribosome recruitment in the RNA-tethering format, we performed the experiments in the presence of Hipp, a small molecule that binds to the eIF4A CTD and inhibits its RNA binding activity by locking eIF4A in a closed conformation (34,35). Translation stimulation by λ-4GIm, λ-4E and λ-4E3 was inhibited by Hipp (34), indicating that the observed response is eIF4A-dependent. Mutations that abolish eIF4E:eIF4G interaction (eIF4E(W73A) and eIF4E(G139D)) (36) were engineered into the analogous positions in eIF4E3 (W85A and G148D) and were found to blunt activity in this assay—suggesting that the observed stimulation by λ-4E3 was also eIF4G-dependent (Figure 3A). Fusing λN to eIF4E2 (4EHP) produced a recombinant protein with no activity in this assay (Figure 3A). PABP also interacts with eIF4G (and the mRNA poly(A) tail) and serves to hold mRNAs in a closed loop configuration, a feature associated with stimulation of translation initiation (7). Even so, PABP did not stimulate FF expression when fused to λN. All constructs expressed proteins of the correct molecular mass (Figure 3B). λ-4E and λ-4E3 were able to interact with eIF4GI, but neither of the two eIF4E3 mutants ((W85A) or (G148)) we tested could (Figure 3C). Taken together, these results indicate that eIF4E3, but not eIF4E2 (4EHP), can potently stimulate translation initiation in a manner that is eIF4G- and eIF4A-dependent.

We also sought to determine if λ-4A fusions could function in our assay (Figure 3D). Both λ-4A1 and λ-4A2 were capable of stimulating translation, but not nearly as robustly as λ-4GIm (Figure 3D). λ-eIF4A3, a paralog that forms a critical component of the exon junction complex, was inactive in this assay. A previously described eIF4A mutant (which we named λ-4A1^Q^) containing four amino acid changes (D265R, E268K, D296A and T298K) that showed impaired ability to interact with eIF4G was also tested (37). We found that λ-4A1^Q^ was unable to stimulate translation, indicating that the results obtained with λ-4A1 and λ-4A2 are eIF4G-dependent (Figure 3D). All constructs expressed proteins of the expected molecular mass (Figure 3E). Whereas endogenous eIF4A1 was solely cytoplasmic, a small proportion of λ-4A1, and λ-4A2 was present in the nucleus while ∼50% of recombinant λ-4A3 was cytoplasmic (Figure 3F). These findings indicate that the failure of λ-4A3 to show activity in the RNA-tethering assay cannot be attributed to its absence from the cytoplasm.

### eIF4G:eIF4A obligate dimers and translation initiation

**Figure 4.**
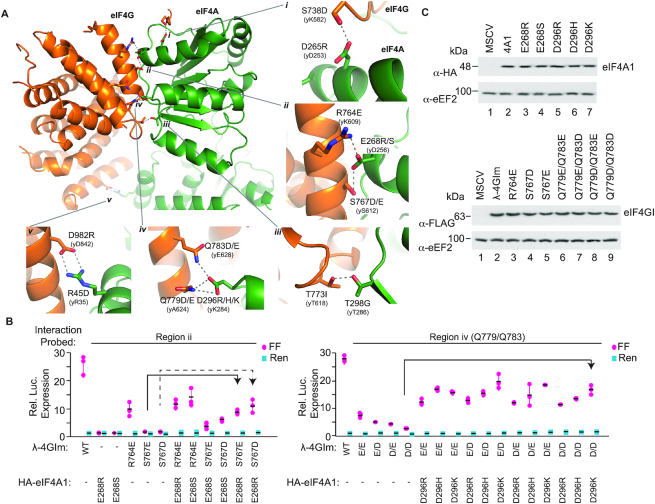
Structure-guided approach to generating orthogonal eIF4G:eIF4A pairs. (**A**) A model of the interface between human eIF4A1:eIF4GI was created by transposing the human sequence onto the yeast (y) structure (38) (PDB accession number 2VSO) (38). Overview of the yeIF4G:eIF4A complex with human residues modelled at the interface. ***i*.** Residue pair eIF4GI(S738):eIF4A1(D265) forms a putative interaction in human. The identity and numbering in yeast homologs of the relevant residues are indicated in parenthesis. The amino acids targeted in human eIF4A1, as well as corresponding mutations made, are indicated. Note that the side chain of yK582 is disordered. ***ii*.** eIF4GI residues R764 and S767 form a putative interaction with eIF4A1 residue E268 in human. ***iii*.** eIF4GI residues T773 and eIF4A1 residue T298 form a putative interaction in human. ***iv*.** eIF4GI residue Q779 and/or eIF4GI residue Q783 forms a putative interaction with eIF4A1 D296 in human. ***v*.** eIF4GI residues D982 and eIF4A1 residue R45 form a putative interaction in human. (**B**) Luciferase production following co-expression of the indicated constructs in HEK293T cells with 3xBBox mRNA. Arrows refer to the increase in signal obtained with the obligate dimer pair compared to signal obtained with only the eIF4G mutant—only the largest increase is denoted in this way. Values are normalized to cells having received empty MSCV expression vector. *n* = 3, ± SD. (**C**). Western blot documenting expression levels of recombinant fusions in transfected HEK293T cells.

In principle, different eIF4F paralogs are expected to be found in mammals – each containing either eIF4A1 or eIF4A2 and eIF4GI or eIF4GII. We therefore assessed if all four different eIF4A:eIF4G combinations could participate in ribosome recruitment. The report of yeast eIF4A:eIF4G co-complex crystal structures (38) prompted us to investigate if we could use this information to develop obligate eIF4A:eIF4G dimers. A number of contact sites are apparent between yeast eIF4G and eIF4A from inspection of the co-complex structure. Transposition of the human sequences onto the yeast structure suggested conservation of five interactions (Figure 4A, labeled i–v), which with human numbering and sequence are (i) eIF4G(S738) with eIF4A(D265), (ii) eIF4G(R764) and eIF4G(S767) with eIF4A(E268), (iii) eIF4G(T773) with eIF4A(T298), (iv) eIF4G(Q783) and eIF4G(Q779) with eIF4A(D296), and (v) eIF4G(D982) with eIF4A(R45) (Figure 4A, Supplementary Figure S4A). We undertook to engineer complementary paired changes at each of these locations in eIF4G and eIF4A in an attempt to generate obligate partners while minimizing interactions with endogenous wild-type partners.

Our strategy was to initially identify λ-4GIm mutants showing a strong reduction in activity in the tethering assay when expressed on their own. This first pass screen served to identify mutants that nominally interacted with endogenous wt eIF4A or had lost activity. To eliminate the latter class of mutants, we then measured the activity of λ-4GIm constructs when co-expressed with their corresponding HA-tagged eIF4A paired mutant in the tethering assay (Figure 4B and Supplementary Figure S4B, C). Our results indicated that (a) λ-4GIm(S738D) [region i] and (b) λ-4GIm(Q779E) or λ-4GIm(Q783E) [region iv] mutants still retained the ability to stimulate BBox-dependent translation (although to varying extents) in the absence of an obligate eIF4A1 partner and that this was not significantly stimulated upon co-expression of HA-4A1(D265R) or HA-4A1(D296R), respectively (Supplementary Figure S4B). λ-4GIm(D982R) [region v] was not active in the tethering assay nor did co-expression of HA-4A1(R45D) lead to translation stimulation (Supplementary Figure S4B). λ-4GIm(T773I) [region iii] was also not active in the tethering assay, and only slight stimulation of translation was observed upon co-expression of HA-4A1(T298G) (Supplementary Figure S4B). These mutants were therefore not pursued any further.

Constructs λ-4GIm(S767D) and λ-4GIm(S767E) [region ii] did not activate BBox-dependent-translation on their own, but when co-expressed with HA-4A1(E268S) or HA-4A1(E268R), stimulation was observed and the largest effect was imparted by HA-4A1(E268R) (Figure 4B, left panel). We also revisited region iv by creating double mutants at eIF4GI residues 779 and 783 and several of these showed translation stimulation when paired with appropriate HA-eIF4A1 partners (Figure 4B, right panel): on their own, all 779/783 mutants had diminished activation, and stimulation was observed when combined with any of three eIF4AI mutants at D296 (D296R/H/K)—with the best stimulation observed with the 4G(Q779D/Q783D) and 4A(D296K) pair (Figure 4B). We confirmed similar expression of all tested constructs [all eIF4A1 and eIF4GI constructs are HA and FLAG tagged, respectively] (Figure 4C).

**Figure 5.**
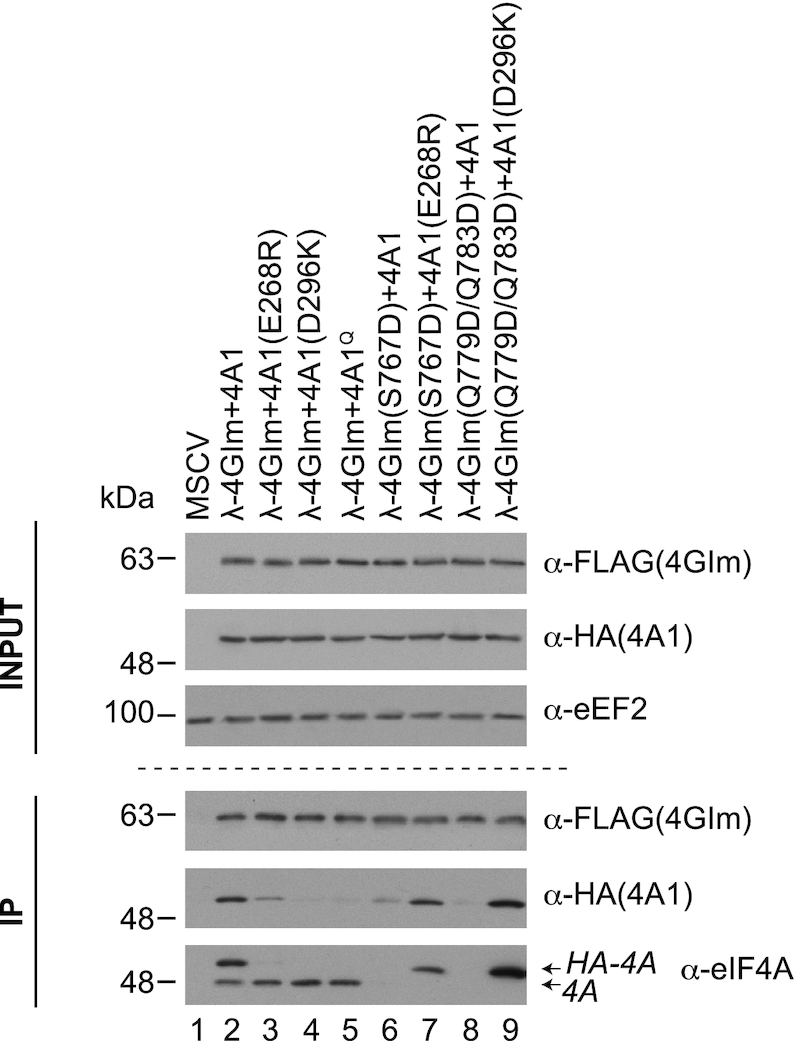
Obligate dimer interaction. Following transfection of HEK293T cells with the indicated expression vectors, cell extracts were prepared 48 h later and used in immunoprecipitations reactions with an anti-FLAG antibody. Following SDS-PAGE, Western blots were undertaken with antibodies shown to the right. Recombinant 4GIm and eIF4A1 proteins are FLAG- and HA-tagged, respectively.

We confirmed the ability of region ii and iv mutants to interact *in cellula* using immunoprecipitation assays (Figure 5). We observed that interaction of HA-tagged eIF4A1(E268R), eIF4A1(D296K) and eIF4AI^Q^ mutants with FLAG-tagged λ-4GIm was significantly reduced, compared to HA-tagged wt eIF4A1 (Figure 5, compare lanes 3–5 to 2). Constructs λ-4GIm(S767D) and λ-m4GI(Q779D/Q783D) failed to interact with endogenous wt eIF4A1, but were able to interact with eIF4A1(E268R) and eIF4A1(D296K), respectively—consistent with these having altered eIF4A1 binding specificity (compare lanes 7 and 9 to 6 and 8, respectively).

**Figure 6.**
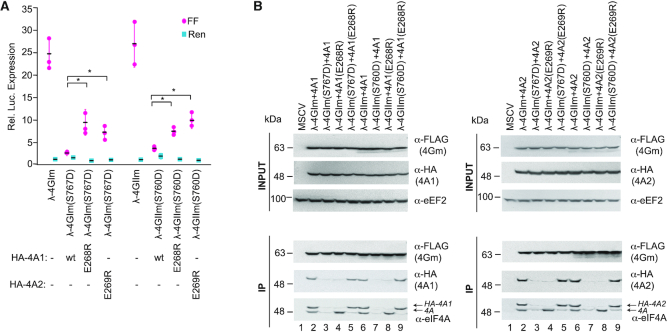
Stimulation of translation by eIF4GI(S767D), eIF4GII(S760D), eIF4A(S268R) and eIF42(S269R) obligate dimer combinations. (**A**) Stimulation, relative to MSCV expression controls, obtained upon transfection of 3×BBox-FF and HCV-Ren mRNA into HEK293T cells. *n* = 3, ±SD. *P < 0.01. (**B**) Anti-FLAG immunoprecipitations of the indicated λN-fusions followed by Western blotting with antibodies indicated to the right of each panel. λ-4G and eIF4A constructs are FLAG and HA tagged, respectively.

We then engineered the 4GI(S767D) mutation into the eIF4GII backbone, to yield 4GII(S760D). We also generated an eIF4A2 mutant corresponding to HA-eIF4A1(E268R), to yield HA-eIF4A2(E269R). These different obligate dimer pairs were tested for their ability to stimulate translation (Figure 6A). All obligate dimer combinations stimulated translation of the 3xBBox mRNA reporter. The ability of each obligate dimer pair to interact with each other was confirmed in immunoprecipitation assays (Figure 6B). (We note the presence of a faster migrating eIF4GIIm product which we suspect to be a cleavage product arising near the CTD since this polypeptide retains the NTD FLAG tag.)

**Figure 7.**
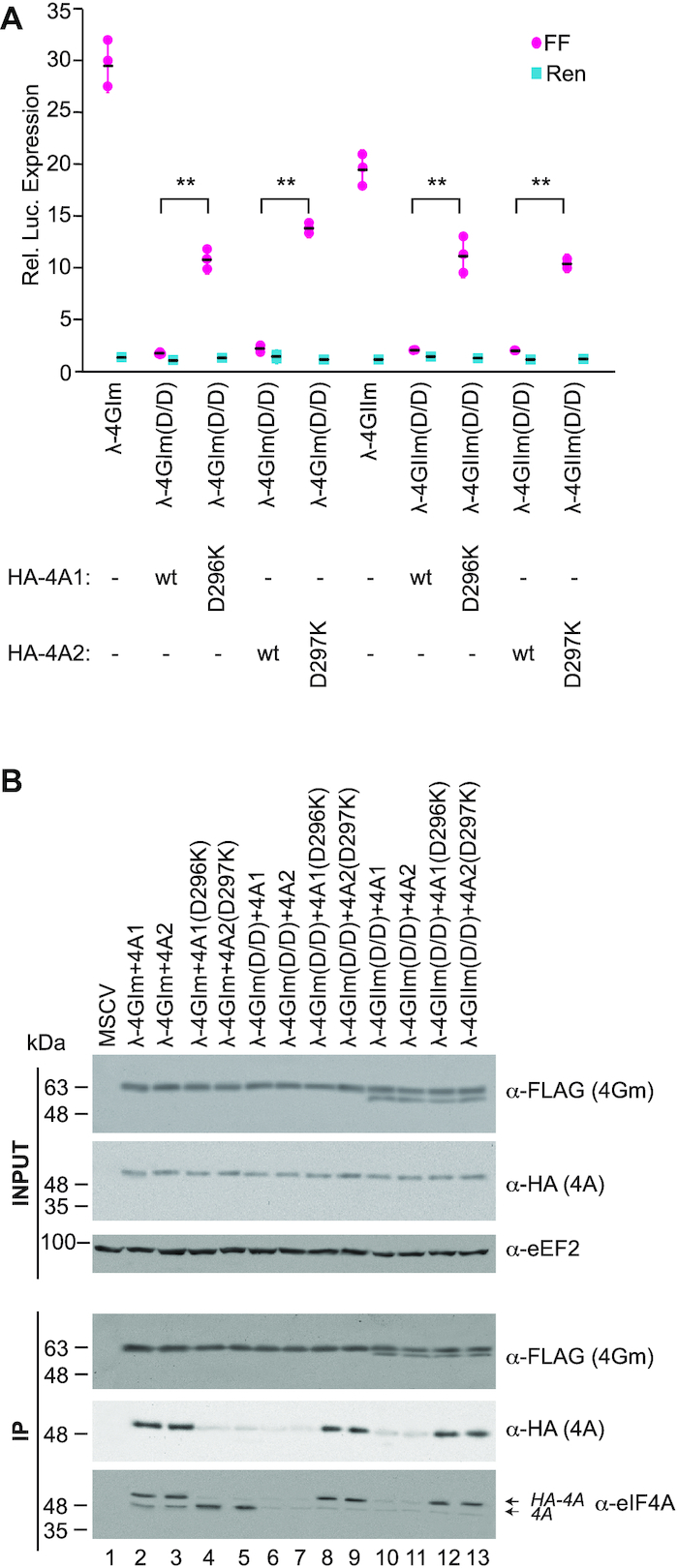
Stimulation of translation by eIF4G:eIF4A obligate dimer combinations. (**A**) Stimulation, relative to MSCV expression controls, obtained upon transfection of the indicated expression constructs and 3×BBox-FF and HCV-Ren mRNA into HEK293T cells. *n* = 3, ± SD. **P< 0.01. (**B**). Anti-FLAG immunoprecipitations of the indicated λN-fusions followed by Western blotting with antibodies indicated to the right of each panel. λ-eIF4G and eIF4A constructs are FLAG and HA tagged, respectively.

We then used a second set of obligate dimers to confirm and extend these results. Here, we engineered the 4GI(Q779D/Q783D) mutations into eIF4GII and the 4A1(D296K) change into eIF4A2, yielding eIF4A2(297K) (Figure 7A). The λ-4GIm double mutant (Q779D/Q783D) was only capable of stimulating translation when eIF4A1(D296K) or eIF4A2(D297K) were present (Figure 7A). The same was true for λ-4GIIm(Q772D/Q776D). Immunoprecipitation experiments demonstrated the expected interactions between all obligate dimers (Figure 7B). Taken together, these experiments demonstrate that all eIF4G/eIF4A combinations are competent for ribosome recruitment and translation initiation.

We took advantage of the obligate dimer configuration to probe for activities of eIF4A1 required to recruit ribosomes. The largest contiguous span of sequence diversity between eIF4A1 and eIF4A2 resides at the N-terminus of the proteins (Supplementary Figure S5A). Deletion of the first 16 and 17 amino acids from eIF4A1 and eIF4A2, respectively, had no impact on translation stimulation activity (Supplementary Figure S5b). Pause *et al.* (39,40) have described a number of eIF4A1 mutants that impact on different activities of eIF4A1.The chosen mutants affected various activities of eIF4A1 and are summarized in Supplementary Figure S6A. Four mutants (DQAD, R362K, R365K, PRRVAA) were reported to be devoid of helicase activity and also showed impaired RNA binding and ATPase activity to varying extents. One mutant, DEAH, was reported to have elevated ATP binding, ATPase, and RNA binding activity, yet only had 10% helicase activity compared to wt eIF4A1 (39). The PRRVAA mutant has also been reported to harbor dominant-negative activity by inhibiting eIF4F–mRNA interaction (41). These mutations were engineered in the context of the D296K obligate partner. With the exception of the DEAH mutant, all other mutants failed to stimulate translation in the tethering assay (Supplementary Figure S6B, C). Although we are unable to assess if the activity observed with this mutant relies on the residual weak helicase activity or elevated ATPase and RNA binding, clearly impairment of RNA binding and lack of helicase activity (R362K, R365K) is sufficient to abolish ribosome recruitment.

### Tripartite extended tethering assay

**Figure 8.**
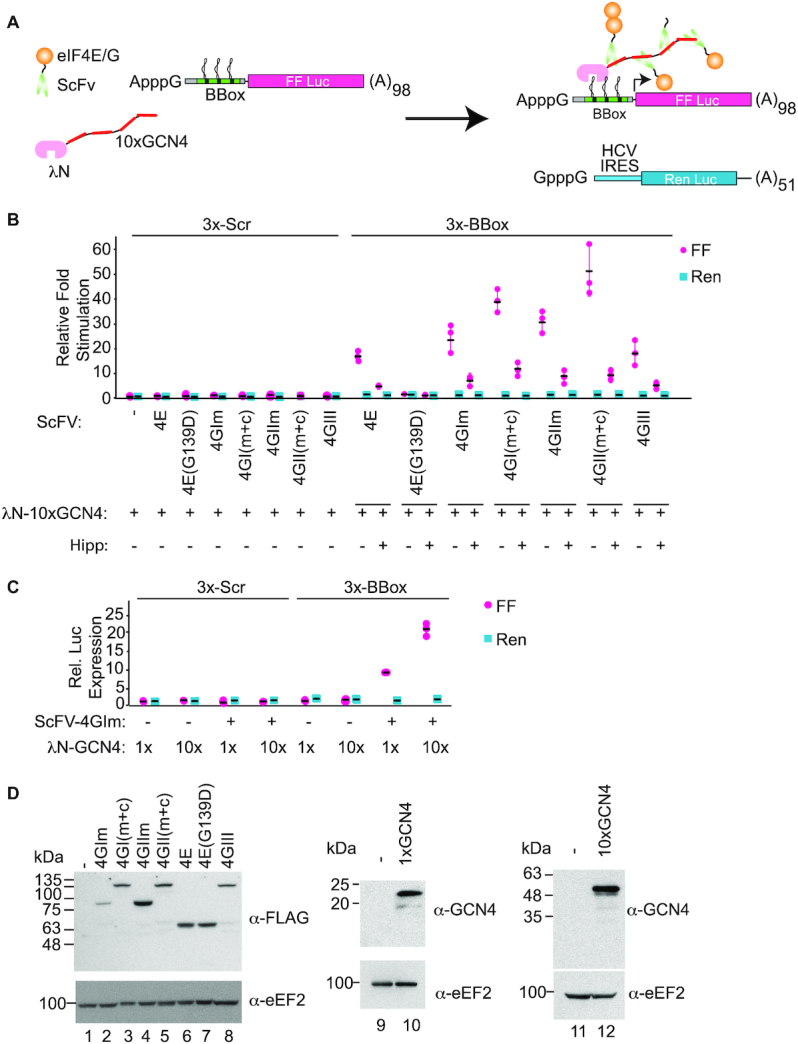
Tripartite tethering assay. (**A**) Schematic diagram of tripartite tethering assay. A single chain antibody recognizing the GCN4 epitope is fused to eIF4E or eIF4G domains (ScFv-4E or ScFv-4G). Ten repeats of the GCN4 epitope are fused to λN to generate the bridging molecule, λN-10xGCN4. (**B**) Stimulation, relative to controls, obtained upon transfection of 3×Scr-FF or 3×BBox-FF and HCV-Ren mRNA into HEK293T cells. *n* = 3, ±SD. (**C**) Translation stimulation of 3xBBox-FF and HCV-Ren mRNAs relative to control in the presence of λN-1×GCN4 or λN-10×GCN4 when co-expressed with ScFV-4GIm. *n* = 3, ± SD. (**D**). Western blotting of the ScFv- and λN-GCN4 fusion constructs using the indicated antibodies.

As a prelude to developing a more modular system by which a ribosome recruiting activity could be delivered to a specific mRNA site, we were inspired by the SunTag system (42) and built a λN-10xGCN4 epitope fusion capable of recruiting chimeras in which an anti-GCN4 single chain binding antibody (ScFv) is fused to eIF4G or eIF4E (Figure 8A). Using A-capped 3×BBox-FF mRNA, robust stimulation was observed with eIF4E, eIF4GIII, and the eIF4GI/IIm and eIF4GI/II(m+c) domains (Figure 8B). The eIF4E(G139D) mutant that is incapable of interacting with eIF4G was unable to stimulate expression in this setting (Figure 8B). The stimulation was eIF4A-dependent as Hipp treatment lead to a significant reduction in expression. No stimulation was detected when the 3×Scr-FF reporter was used (Figure 8B) or when the 10×GCN4 fusion was omitted from the transfections (Supplementary Figure S7). Furthermore, λN fusions containing only one GCN4 epitope were able to stimulate translation to levels that were ∼50% of those obtained with 10xGCN4 (Figure 8C). All constructs expressed recombinant proteins (Figure 8D). These experiments demonstrate that eIF4E or eIF4G need not be directly bound to the mRNA template to mediate ribosome recruitment and demonstrate the possibility of *in trans* ribosome recruitment and translation initiation.

## DISCUSSION

The tethering assay, as shown by Hentze and co-workers (21,22) and adopted herein, is quite powerful for teasing out ribosome recruitment activity independent of translation initiation factor RNA binding activity. One parameter that appears to impact on sensitivity is the number of engineered BBoxes, with 3× and 6× BBox repeats yielding responses more robustly than a single BBox, a phenomenon also noted by De Gregorio *et al.* (21) (Figure 1). We saw little differences when the BBoxes were spaced 26 versus 52 nts apart (Figure 1 and Supplementary Figure S2). The increase in activity observed with multiple BBoxes is more difficult to rationalize but may be due to cooperative assembly and stabilization of the λ-4GIm fusions, which in turn could increase the likelihood of a productive ribosome recruitment event occurring. Alternatively, cooperative binding of downstream co-factors (e.g. free eIF4A or eIF4B) may be promoted by the presence of multiple, anchored λ-4GIm/eIF4A molecules.

Our results indicate that eIF4GI, eIF4GII, and eIF4GIII can function to recruit ribosomes *in vivo*. These results are at odds with reports that over-expression of eIF4GIII inhibits cap-dependent and cap-independent translation (43,44), but are consistent with the reported stimulatory role of eIF4GIII in supporting synthesis of factors required for ES cell differentiation (9). It may be that under conditions where eIF4GIII is over-expressed, this leads to sequestration of interacting partners (e.g. eIF4A, eIF2β, eIF3) by the recombinant protein and generates non-productive complexes—resulting in inhibition of translation. The middle core domains of eIF4GI and eIF4GII appear sufficient for ribosome recruitment, although the presence of the C-terminal domain clearly increased translation by ∼30% (Figures 2C, 8B and Supplementary Figure S3). This may be the result of better eIF4A1/2 recruitment since this portion of eIF4G contains an additional binding site for eIF4A. Our results defining the mTSD of eIF4GIm (Supplementary Figure S3) mapped the minimal region required to yield activity similar to full-length eIF4GIm to residues 653 and 1053. These results are consistent with a previous study indicating the presence of an eIF4A interacting domain within eIF4GIm that spans amino acids 674–1079 (45). A deletion that removed the 33 amino acids from the N-terminal domain (λ-719–1131) showed a significant reduction in activity (compare to λ-686–1131) (Supplementary Figure S3) indicating that we are close to the N-terminal boundary. Two subdomains within eIF4G required for eIF3c/d and eIF3e interaction that encompass residues 1011–1051 and 1052–1104 have been described (5). The eIF3e subdomain was identified in a tethering assay as being required to achieve full translation stimulation activity, but was defined in the absence of the eIF4A interacting domain (5). Our results would suggest that eIF3e interaction may not be necessary for ribosome recruitment when the eIF4A interacting domain is present.

We capitalized on yeast eIF4G:eIF4A structural information to design and test mutations of interacting amino acids that were also predicted to be present in the mammalian homologs (38). This lead to the identification of mutations from two regions that generated obligate dimers: (i) eIF4GIm(S767D) with eIF4A1(E268R) or eIF4A2(E269R), (ii) eIF4GIIm(S760D) with eIF4A1(E268R) or eIF4A2(E269R) as well as (iii) eIF4GIm (Q779D/Q783D) or eIF4GIIm(Q772D/Q776D) and eIF4AI(D296K) or eIF4A2(D297K) (Figure 4; regions ii and iv). We used these obligate dimers to demonstrate that all permutations of eIF4GI/II and eIF4A1/2 could recruit ribosomes and stimulate translation. Defining differences in activity or nuances in mRNA targeting among the eIF4F complexes will be a future challenge that should be aided by the obligate dimers that we have defined herein.

The ability to generate obligate dimers between eIF4A and eIF4G enabled us to test the ability of different eIF4A1 mutants to participate in the ribosome recruitment process (Supplementary Figure S6A). Each mutant showed impairment in more than one activity making it difficult to attribute the loss of translation to a single enzymatic function, but the results with R362K and R365K showing impaired RNA binding and helicase activity indicate that these properties are essential for initiation. Recent experiments have shown that eIF4A plays a role beyond resolving secondary structure in the 5′ leader region, as it also functionally interacts with the pre-initiation complex (PIC) to promote mRNA-ribosome recruitment (46,47), and in the mammalian setting this activity has been shown to be independent of eIF4A helicase activity (46). Experiments with the obligate dimers are performed in the presence of wt eIF4A1 (which may still associate with PICs) indicating that the inhibition on translation observed with the eIF4A1 mutants in the current study is primarily due to impaired activity of the eIF4G/eIF4A dimer to properly remodel the mRNA for the incoming PIC or due to impaired eIF4G/eIF4A-PIC recruitment. The ability of the DEAH mutant to efficiently recruit ribosomes with only 10% wt helicase activity, indicates that this activity is not limiting for PIC recruitment by eIF4G.

We also tested the ability of other components of the translation initiation pathway to recruit ribosomes and stimulate translation of BBox-containing reporters. We note that the inability of λ-4E2 (4EHP) to function in this assay (Figure 3) is consistent with its role as a repressor of translation and its inability to interact with eIF4G (17). In contrast, we found eIF4E3 to be a potent stimulator of translation initiation—an activity that was dependent on eIF4A and interaction with eIF4G (Figure 3). These results are at odds with the finding that ectopic over-expression of eIF4E3 decreased expression of target mRNAs (VEGF, c-Myc, Cyclin D1, NBS1) in cells (18). It remains possible that depending on cellular context and expression levels, eIF4E3 might stimulate or inhibit translation of select mRNAs. Expression of eIF4E3 mRNA is quite low or undetectable in many tissues (17) and so the extent and conditions under which eIF4F complexes harboring eIF4E3 mold the cellular proteome will be a topic for future studies.

We have probed for the ability of eIF3d and eIF3l to stimulate translation initiation in the tethering assay since these have been previously attributed cap binding activity (31,32). In our hands, these factors showed no activity and this may indicate that the tethering assay is not faithfully capturing all features required for these proteins to interact with the mRNA at the cap structure. The same was true with a λ-PABP fusion (Figure 3B), which we tested because of its association with eIF4G and which failed to recruit ribosomes. However, we urge caution in interpretation of these preliminary results.

With the presence of two cap binding proteins that stimulate translation, eIF4E1 and eIF4E3, our results suggest the existence of eight different eIF4F complexes that could function to recruit ribosomes and stimulate translation initiation. Solely on the basis of BBox-mediated translation stimulation, we show that in cells, all of these complexes function at similar efficiency. This may not reflect the canonical recruitment process that occurs in cells as the tethering assay completely bypasses the initial binding of translation factors to the cap. Our results do reveal that from a mechanistic point of view, all components of the eIF4F complex can recruit ribosomes on an mRNA template using either paralogs of eIF4G and eIF4A. Based on this, it remains to be determined why these redundant proteins exist in cells. eIF4A2 expression is known to be stimulated when eIF4A1 levels are reduced, but this compensatory effect cannot rescue cell growth defect associated with eIF4A loss (48). A possibility is that these factors may be expressed differently or have contrasting activities depending on mRNA templates, cell types or stages of cellular development.

We were able to demonstrate that direct tethering of eIF4G or eIF4E to the reporter mRNA is not required to achieve translation stimulation but could be meditated by an intermediate bridging molecule (Figure 8, e.g. λ-10xGCN4). It has not escaped our attention that these experiments suggest that a bifunctional small molecule that interacts with a specific RNA feature and that is also capable of recruiting eIF4GI/II could be used to stimulate translation initiation on a given RNA template at a pre-specified address. Our work sets the stage for synthetic approaches to be developed by which translation of specific mRNAs can be manipulated.

## Supplementary Material

gkaa646_Supplemental_File
