# Supplementary material for: RNA-tethering assay and eIF4G:eIF4A obligate dimer design uncovers multiple eIF4F functional complexes
Source: Nucleic Acids Res. 2020 Aug 4;48(15):8562–75. doi: 10.1093/nar/gkaa646 (PMC7470955; doi:10.1093/nar/gkaa646)
Supplement: gkaa646_Supplemental_File [file gkaa646_supplemental_file.pdf]

Supplemental Figure Legends

Figure S1. Nucleotide sequence information of luciferase reporters used in this study.

Figure S2. Translation of the indicated Scr- or BBox-containing mRNAs in Krebs-2 extracts. **a.** Average raw light units of the indicated m_7G-capped Scr- or BBox-containing FF-BBox-Ren bicistronic mRNAs when translated *in vitro* in K-2 extract at 10 μg/ml. n = 3, ± SD. **b.** Luciferase production following *in vitro* translation of 10 μg/ml of the indicated A-capped FF and HCV-Ren mRNA in K-2 or K-2/λ–4GIm extracts. Values are normalized to the Scr control mRNAs of the same length. n = 3, ± SD.

Figure S3. Fine deletion mapping of eIF4GI mTSD. **a.** Schematic diagram of eIF4GIm deletions tested herein. The α-helical regions, as defined in Marcotrigiano et al. (29) and labelled Ia - Vb within the HEAT/MIF4G region, are indicated by solid black lines. **b.** Stimulation relative to MSCV expression controls, obtained upon transfection of A-capped 3xBBox-FF and HCV-Ren mRNA into HEK293T cells. n = 3, ± SD. **c.** Western blot documenting expression of λ-4GIm deletions in transfected HEK293T cells.

Figure S4 Characterizing orthogonal eIF4G:eIF4A pairs. **a.** Conservation of the interface regions between human and yeast eIF4G:eIF4A showing amino acids selected for mutagenesis. M-1 and M-4 refer to two of four motifs identified by Imataka and Sonenberg (45) as being critical for eIF4A interaction. **b.** Stimulation relative to MSCV expression controls, obtained upon transfection of 3xBBox-FF and HCV-Ren mRNA with the indicated expression vectors into HEK293T cells. n = 3, ± SD. **c.** Western blot documenting expression of HA-4A1 and λ-4GIm mutants in transfected HEK293T cells.

Figure S5. a. Needleman-Wunsch alignment performed between human eIF4A1 (NP_001407) and human eIF4A2 (NP_001958) at https://blast.ncbi.nlm.nih.gov/Blast.cgi. The yellow box highlights the amino acids deleted to generate the N-terminal eIF4A mutations. **b.** Stimulation, relative to MSCV expression controls, obtained upon transfection of 3xScr-FF (first lane) or 3xBBox-FF and HCV-Ren mRNA into HEK293T cells. n = 3, ± SD.

Figure S6. Activity of eIF4A1 mutants in the obligate dimer context. **a.** Table summarizing biochemical information on the various mutants tested in this study. Data is taken from Pause et al. (40,41) and values represent % activity relative to wild-type protein. **b.** Stimulation, relative to MSCV expression controls, obtained upon transfection of the indicated expression constructs and 3xBBox-FF and HCV-Ren mRNA into HEK293T cells. n = 4, ± SD. ns, p=0.75 **c.** Western blot of recombinant HA-tagged eIF4A1 mutants in HEK293T cells.

Figure S7. Activity of different ScFv-fusion construct towards 3xBBox-FF and HCV-Ren mRNAs in HEK293T cells in absence of λN-10xGCN4. n = 3 ± SD.

FIGURE S1
Monocistronic reporter

T3-1XBBoxes-FF-pA:

T3 promoter
AATTAACCCTCACTAAAGGGAACAAAAGCTGGTACCGGGCCCCCCCCTCGAGCATCCACTTTGCCTTTC
Bbox **FF start**
TCTCC**GGGCCCTGAAGAAGGGCCC**CTAGAGTACTTAATACGACTCACTATAGCTCGAGTCATGGAAGA…

T3-1xScr_Luc_pA+

T3 promoter
AATTAACCCTCACTAAAGGGAACAAAAGCTGGTACCGGGCCCCCCCCTCGAGCATCCACTTTGCCTTTC
FF start
TCTCC**GAGCTGAACGGCGCCAGCG**CTAGAGTACTTAATACGACTCACTATAGCTCGAGTCATGGAAGA…

T3-3XBBoxes-FF-pA:

T3 promoter
AATTAACCCTCACTAAAGGGAACAAAAGCTGGTACCGGGCCCCCCCCTCGAGCATCCACTTTGCCTTTC
Bbox ***Bbox***
TCTCC**GGGCCCTGAAGAAGGGCCC**CAACAATCCACTTTGCCTTTCTCTCC**GGGCCCTGAAGAAGGGCCC**
Bbox
TCGTTCCACTGAGCGTCAGAAACAAC**GGGCCCTGAAGAAGGGCCC**CTAGAGTACTTAATACGACTCACT
FF start
ATAGCTCGAGTCATGGAAGAC…

T3-3xScr_Luc_pA+

T3 promoter
AATTAACCCTCACTAAAGGGAACAAAAGCTGGTACCGGGCCCCCCCCTCGAGCATCCACTTTGCCTTTC

TCTCC**GAGCTGAACGGCGCCAGCG**CAACAATCCACTTTGCCTTTCTCTCC**GAGCTGAACGGCGCCAGCG**

TCGTTCCACTGAGCGTCAGAAACAAC**GAGCTGAACGGCGCCAGCG**CTAGAGTACTTAATACGACTCACT
FF start
ATAGCTCGAGTCATGGAAGAC…

T3-6XBBoxes-FF-pA:

T3 promoter ***BBox***
AATTAACCCTCACTAAAGGGAACAAAAGCTGGTACCGGGCCCCCC**gggccctgaagaagggccc**caaca
BBox
Atccactttgcctttctctcc**gggccctgaagaagggccc**tcgttccactgagcgtcagaaacaac**ggg**
BBox ***BBox***
ccctgaagaagggcccacagctgtctagcagttctaatcttt**gggccctgaagaagggcccc**aacaatc
BBox
cactttgcctttctctcc**gggccctgaagaagggccc**tcgttccactgagcgtcagaaacaac**gggccc**
Bbox **FF start**
tgaagaagggcccCTAGAGTACTTAATACGACTCACTATAGCTCGAGTCATGGAAGAC…

T3-6xScr_Luc_pA+

T3 promoter
AATTAACCCTCACTAAAGGGAACAAAAGCTGGTACCGGGCCCCCCCCTCGAGCATCCACTTTGCCTTTC

GAGCTGAACGGCGCCAGCGCAACAATCCACTTTGCCTTTCTCTCC**GAGCTGAACGGCGCCAGCG**TCGTT

CCACTGAGCGTCAGAAACAAC**GAGCTGAACGGCGCCAGCG**ACAGCTGTCTAGCAGTTCTAATCTTT**GAG**

CTGAACGGCGCCAGCGCAACAATCCACTTTGCCTTTCTCTCC**GAGCTGAACGGCGCCAGCG**TCGTTCCA

CTGAGCGTCAGAAACAAC**GAGCTGAACGGCGCCAGCG**CTAGAGTACTTAATACGACTCACT

FF start →

ATAGCTCGAGTCATGGAAGAC…

Bicictronic reporter:

pKS/FF/1xSCR/Ren:

gtggcacttttcggggaaatgtgcgcggaacccctatttgtttatttttctaaatacattcaaatatgt
atccgctcatgagacaataaccctgataaatgcttcaataatattgaaaaaggaagagtatgagtattc
aacatttccgtgtcgcccttattcccttttttgcggcattttgccttcctgtttttgctcacccagaaa
cgctggtgaaagtaaaagatgctgaagatcagttgggtgcacgagtgggttacatcgaactggatctca
acagcggtaagatccttgagagttttcgccccgaagaacgttttccaatgatgagcacttttaaagttc
tgctatgtggcgcggtattatcccgtattgacgccgggcaagagcaactcggtcgccgcatacactatt
ctcagaatgacttggttgagtactcaccagtcacagaaaagcatcttacggatggcatgacagtgagaa
ttatgcagtgctgccataaccatgagtgataacactgcggccaacttacttctgacaacgatcggagga
ccgaaggagctaaccgcttttttgcacaacatgggggatcatgtaactcgccttgatcgttgggaaccg
gagctgaatgaagccataccaaacgacgagcgtgacaccacgatgcctgtagcaatggcaacaacgttg
cgcaaactattaactggcgaactacttactctagcttcccggcaacaattaatagactggatggaggcg
gataaagttgcaggaccacttctgcgctcggcccttccggctggctggtttattgctgataaatctgga
gccggtgagcgtgggtctcgcggtatcattgcagcactggggccagatggtaagccctcccgtatcgta
gttatctacacgacggggagtcaggcaactatggatgaacgaaatagacagatcgctgagataggtgcc
tcactgattaagcattggtaactgtcagaccaagtttactcatatatactttagattgatttaaaactt
catttttaatttaaaaggatctaggtgaagatcctttttgataatctcatgaccaaaatcccttaacgt
gagttttcgttccactgagcgtcagaccccgtagaaaagatcaaaggatcttcttgagatcctttttt
ctgcgcgtaatctgctgcttgcaaacaaaaaaaccaccgctaccagcggtggtttgtttgccggatcaa
gagctaccaactctttttccgaaggtaactggcttcagcagagcgcagataccaaatactgtccttcta
gtgtagccgtagttaggccaccacttcaagaactctgtagcaccgcctacatacctcgctctgctaatc
ctgttaccagtggctgctgccagtggcgataagtcgtgtcttaccgggttggactcaagacgatagtta
ccggataaggcgcagcggtcgggctgaacggggggttcgtgcacacagcccagcttggagcgaacgacc
tacaccgaactgagatacctacagcgtgagctatgagaaagcgccacgcttcccgaagggagaaaggcg
gacaggtatccggtaagcggcagggtcggaacaggagagcgcacgagggagcttccagggggaaacgcc
tggtatctttatagtcctgtcgggtttcgccacctctgacttgagcgtcgatttttgtgatgctcgtca
ggggggcggagcctatggaaaaacgccagcaacgcggcctttttacggttcctggccttttgctggcct
tttgctcacatgttctttcctgcgttatcccctgattctgtggataaccgtattaccgcctttgagtga
gctgataccgctcgccgcagccgaacgaccgagcgcagcgagtcagtgagcgaggaagcggaagagcgc
ccaatacgcaaaccgcctctccccgcgcgttggccgattcattaatgcagctggcacgacaggtttccc
gactggaaagcgggcagtgagcgcaacgcaattaatgtgagttagctcactcattaggcaccccaggct
ttacactttatgctcccggctcgtatgttgtgtggaattgtgagcggataacaatttcacacaggaaac

T3 Promoter +1

agctatgaccatgattacgccaagcgcgc*AATTAACCCTCACTAAA**G**gg*aacaaaagctgggtaccggg
ccccccctcgaggtcgacggtatcgataagcttgatatcgaattcctgcagcccgggggatctgcgatc

Firefly Luciferase

taagtaagcttggcattccggtactgttggtaaagccaccATGGAAGACGCCAAAAACATAAAGAAAGG
CCCGGCGCCATTCTATCCGCTGGAAGATGGAACCGCTGGAGAGCAACTGCATAAGGCTATGAAGAGATA
CGCCCTGGTTCCTGGAACAATTGCTTTTACAGATGCACATATCGAGGTGGACATCACTTACGCTGAGTA
CTTCGAAATGTCCGTTCGGTTGGCAGAAGCTATGAAACGATATGGGCTGAATACAAATCACAGAATCGT
CGTATGCAGTGAAAACTCTCTTCAATTCTTTATGCCGGTGTTGGGCGCGTTATTTATCGGAGTTGCAGT
TGCGCCCGCGAACGACATTTATAATGAACGTGAATTGCTCAACAGTATGGGCATTTCGCAGCCTACCGT
GGTGTTCGTTTCCAAAAAGGGGTTGCAAAAAATTTTGAACGTGCAAAAAAAGCTCCCAATCATCCAAAA
AATTATTATCATGGATTCTAAAACGGATTACCAGGGATTTCAGTCGATGTACACGTTCGTCACATCTCA
TCTACCTCCCGGTTTTAATGAATACGATTTTGTGCCAGAGTCCTTCGATAGGGACAAGACAATTGCACT
GATCATGAACTCCTCTGGATCTACTGGTCTGCCTAAAGGTGTCGCTCTGCCTCATAGAACTGCCTGCGT
GAGATTCTCGCATGCCAGAGATCCTATTTTTGGCAATCAAATCATTCCGGATACTGCGATTTTAAGTGT
TGTTCCATTCCATCACGGTTTTGGAATGTTTACTACACTCGGATATTTGATATGTGGATTTCGAGTCGT
CTTAATGTATAGATTTGAAGAAGAGCTGTTTCTGAGGAGCCTTCAGGATTACAAGATTCAAAGTGCGCT
GCTGGTGCCAACCCTATTCTCCTTCTTCGCCAAAAGCACTCTGATTGACAAATACGATTTATCTAATTT
ACACGAAATTGCTTCTGGTGGCGCTCCCCTCTCTAAGGAAGTCGGGGAAGCGGTTGCCAAGAGGTTCCA
TCTGCCAGGTATCAGGCAAGGATATGGGCTCACTGAGACTACATCAGCTATTCTGATTACACCCGAGGG
GGATGATAAACCGGGCGCGGTCGGTAAAGTTGTTCCATTTTTTGAAGCGAAGGTTGTGGATCTGGATAC
CGGGAAAACGCTGGGCGTTAATCAAAGAGGCGAACTGTGTGTGAGAGGTCCTATGATTATGTCCGGTTA
TGTAAACAATCCGGAAGCGACCAACGCCTTGATTGACAAGGATGGATGGCTACATTCTGGAGACATAGC
TTACTGGGACGAAGACGAACACTTCTTCATCGTTGACCGCCTGAAGTCTCTGATTAAGTACAAAGGCTA
TCAGGTGGCTCCCGCTGAATTGGAATCCATCTTGCTCCAACACCCCAACATCTTCGACGCAGGTGTCGC
AGGTCTTCCCGACGATGACGCCGGTGAACTTCCCGCCGCCGTTGTTGTTTTGGAGCACGGAAAGACGAT
GACGGAAAAAGAGATCGTGGATTACGTCGCCAGTCAAGTAACAACCGCGAAAAAGTTGCGCGGAGGAGT
TGTGTTTGTGGACGAAGTACCGAAAGGTCTTACCGGAAAACTCGACGCAAGAAAAATCAGAGAGATCCT

Intercistronic

CATAAAGGCCAAGAAGGGCGGAAAGATCGCCGTGTAAttctagtcatccactttgcctttctctc**gagctgaacggcgcca**

Region

Renilla Luciferase

gcgctagagtacttaatacgactcactataggctagccaccATGGCTTCCAAGGTGTACGACCCCGAGCAACGCAAACGC
ATGATCACTGGGCCTCAGTGGTGGGCTCGCTGCAAGCAAATGAACGTGCTGGACTCCTTCATCAACTAC
TATGATTCCGAGAAGCACGCCGAGAACGCCGTGATTTTTCTGCATGGTAACGCTGCCTCCAGCTACCTG
TGGAGGCACGTCGTGCCTCACATCGAGCCCGTGGCTAGATGCATCATCCCTGATCTGATCGGAATGGGT
AAGTCCGGCAAGAGCGGGAATGGCTCATATCGCCTCCTGGATCACTACAAGTACCTCACCGCTTGGTTC
GAGCTGCTGAACCTTCCAAAGAAAATCATCTTTGTGGGCCACGACTGGGGGGCTTGTCTGGCCTTTCAC
TACTCCTACGAGCACCAAGACAAGATCAAGGCCATCGTCCATGCTGAGAGTGTCGTGGACGTGATCGAG
TCCTGGGACGAGTGGCCTGACATCGAGGAGGATATCGCCCTGATCAAGAGCGAAGAGGGCGAGAAAATG
GTGCTTGAGAATAACTTCTTCGTCGAGACCATGCTCCCAAGCAAGATCATGCGGAAACTGGAGCCTGAG
GAGTTCGCTGCCTACCTGGAGCCATTCAAGGAGAAGGGCGAGGTTAGACGGCCTACCCTCTCCTGGCCT
CGCGAGATCCCTCTCGTTAAGGGAGGCAAGCCCGACGTCGTCCAGATTGTCCGCAACTACAACGCCTAC
CTTCGGGCCAGCGACGATCTGCCTAAGATGTTCATCGAGTCCGACCCTGGGTTCTTTTCCAACGCTATT
GTCGAGGGAGCTAAGAAGTTCCCTAACACCGAGTTCGTGAAGGTGAAGGGCCTCCACTTCAGCCAGGAG
GACGCTCCAGATGAAATGGGTAAGTACATCAAGAGCTTCGTGGAGCGCGTGCTGAAGAACGAGCAGTAA
ttctaggtttcaagcttcgggtgggaaaaaaaaaaaaaaaaaaaaaaaaaaaaaaaaaaaaaaaaaaaa
aaaaaaaaaaaaaaaaaaaaaaaaaaaaaaaaaaaaaaaaaaaaaaaaaaaaaaaaaaagggggatcca
ctagttctagagcggccgccaccgcggtggagctccaattcgccctatagtgagtcgtattacgcgcgc
tcactggccgtcgttttacaacgtcgtgactgggaaaaccctggcgttacccaacttaatcgccttgca
gcacatccccctttcgccagctggcgtaatagcgaagaggcccgcaccgatcgcccttcccaacagttg
cgcagcctgaatggcgaatgggacgcgccctgtagcggcgcattaagcgcggagggtgtggtggttacg
cgcagcgtgaccgctacacttgccagcgccctagcgcccgctcctttcgctttcttcccttcctttctc
gccacgttcgccggctttccccgtcaagctctaaatcgggggctccctttagggttccgatttagtgct
ttacggcacctcgaccccaaaaaacttgattagggtgatggttcacgtagtgggccatcgccctgatag
acggtttttcgccctttgacgttggagtccacgttctttaatagtggactcttgttccaaactggaaca
acactcaaccctatctcggtctattcttttgatttataagggattttgccgatttcggcctattggtta
aaaaatgagctgatttaacaaaaatttaacgcgaattttaacaaaatattaacgcttacaatttag

1xBBox:

BBox
…TAAttctagtcatccactttgcctttctctc**gggccctgaagaagggccc**ctagagtacttaatacga
ctcactataggctagccaccATG…

3xScr:

…TAAttctagtcatccactttgcctttctctc**gagctgaacggcgccagcg**caacaatccactttgcct
ttctctcc**gagctgaacggcgccagcg**tcgttccactgagcgtcagaaacaac**gagctgaacggcgcca
gcg**ctagagtacttaatacgactcactataggctagccaccATG…

3xBBox:

BBox
…TAAttctagtcatccactttgcctttctctc**gggccctgaagaagggccc**caacaatccactttgcct
BBox ***BBox***
ttctctcc**gggccctgaagaagggccc**tcgttccactgagcgtcagaaacaac**gggccctgaagaaggg
ccc**ctagagtacttaatacgactcactataggctagccaccATG…

3xBBox*:

BBox
…TAAttctagaattaattaagctgaaccggtattggcgtacactacgattgc**gggccctgaagaagggc
Cc**caacaatccactttgcctttctctcc**gggccctgaagaagggccc**tcgttccactgagcgtcagaaa
BBox
caac**gggccctgaagaagggccc**acctactcagacgcagctcgtgcggcgtcaatacctaggatcaggc
ctggctagccaccATG…

3xBBox-52 Spacing*:
…TAAttctagaattaattaagctgaaccggtattggcgtacactacgattgc**gggccctgaagaagggc
cc**caacaatccactttgcctttctctccacgtactcatcccaactgattctcgg**gggccctgaagaagg
gccc**caatctacggagcgacctgattatcatcgttccactgagcgtcagaaacaac**gggccctgaagaa
gggccc**acctactcagacgcagctcgtgcggcgtcaatacctaggatcaggcctggctagccaccATG…

6xBBox*:

…TAAttctagaattaattaagctgaaccggtattggcgtacactacgattgc**gggccctgaagaagggc
cc**caacaatccactttgcctttctctcc**gggccctgaagaagggccc**tcgttccactgagcgtcagaaa
caac**gggccctgaagaagggccc**acagctgtctagcagttctaatcttt**gggccctgaagaagggccc**c
aacaatccactttgcctttctctcc**gggccctgaagaagggccc**tcgttccactgagcgtcagaaacaa
c**gggccctgaagaagggccc**acctactcagacgcagctcgtgcggcgtcaatacctaggatcaggcctg
gctagccaccATG…

6xBBox-52 Spacing*:
…TAAttctagaattaattaagctgaccggtattggcgtacactacgattgc**gggccctgaagaagggcc**
ccaacaatccactttgcctttctctccacgtactcatcccaactgattctcgg**gggccctgaagaaggg**
ccccaatctacggagcgacctgattatcatcgttccactgagcgtcagaaacaac**gggccctgaagaag**
ggcccacagctgtctagcagttctaatctttattgaggatacgtacactacgattgc**gggccctgaaga**
agggccccaacaatccactttgcctttctctccacgtactcatcccaactgattctcgg**gggccctgaa**
gaagggccccaatctacggagcgacctgattatcatcgttccactgagcgtcagaaacaac**gggccctg**
aagaagggcccacctactcagacgcagctcgtgcggcgtcaatacctaggatcaggcctggctagccac
cATG…

3xScr*:
…TAAttctagaattaattaagctgaccggtattggcgtacactacgattgc**gagctgaacggcgccagc**
gcaacaatccactttgcctttctctcc**gagctgaacggcgccagcg**tcgttccactgagcgtcagaaac
aac**gagctgaacggcgccagcg**acctactcagacgcagctcgtgcggcgtcaatacctaggatcaggcc
tggctagccaccATG…

3xScr-52 Spacing*:
…TAAttctagaattaattaagctgaccggtattggcgtacactacgattgc**gagctgaacggcgccagc**
gcaacaatccactttgcctttctctccacgtactcatcccaactgattctcgg**gagctgaacggcgcca**
gcgcaatctacggagcgacctgattatcatcgttccactgagcgtcagaaacaac**gagctgaacggcgc**
cagcgacctactcagacgcagctcgtgcggcgtcaatacctaggatcaggcctggctagccaccATG…

6xScr*:
…TAAttctagaattaattaagctgaccggtattggcgtacactacgattgc**gagctgaacggcgccagc**
gcaacaatccactttgcctttctctcc**gagctgaacggcgccagcg**tcgttccactgagcgtcagaaac
aac**gagctgaacggcgccagcg**acagctgtctagcagttctaatcttt**gagctgaacggcgccagcg**ca
acaatccactttgcctttctctcc**gagctgaacggcgccagcg**tcgttccactgagcgtcagaaacaac
gagctgaacggcgccagcgacctactcagacgcagctcgtgcggcgtcaatacctaggatcaggcctgg
ctagccaccATG…

6xScr*-52 Spacing:
…TAAttctagaattaattaagctgaccggtattggcgtacactacgattgc**gagctgaacggcgccagc**
gcaacaatccactttgcctttctctccacgtactcatcccaactgattctcgg**gagctgaacggcgcca**
gcgcaatctacggagcgacctgattatcatcgttccactgagcgtcagaaacaac**gagctgaacggcgc**
cagcgacagctgtctagcagttctaatctttattgaggatacgtacactacgattgc**gagctgaacggc**
gccagcgcaacaatccactttgcctttctctccacgtactcatcccaactgattctcgg**gagctgaacg**
gcgccagcgcaatctacggagcgacctgattatcatcgttccactgagcgtcagaaacaac**gagctgaa**
cggcgccagcgacctactcagacgcagctcgtgcggcgtcaatacctaggatcaggcctggctagccac
cATG…

a

b

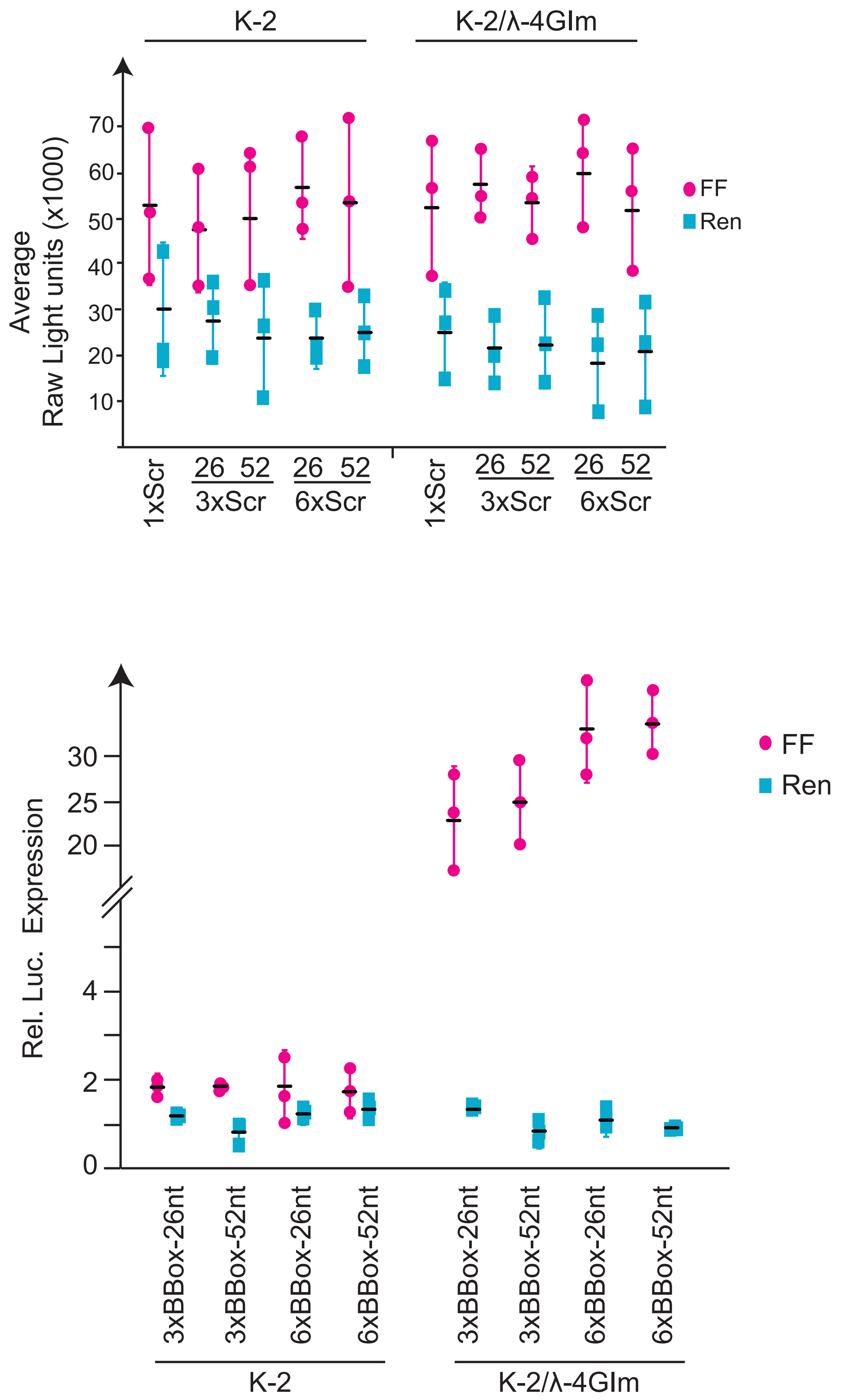
K-2
K-2/λ-4GIm
Average
Raw Light units (x1000)
70
60
50
40
30
20
10
FF
Ren
1xScr
26
52
3xScr
26
52
6xScr
1xScr
26
52
3xScr
26
52
6xScr
Rel. Luc. Expression
30
25
20
4
2
0
FF
Ren
3xBBox-26nt
3xBBox-52nt
6xBBox-26nt
6xBBox-52nt
3xBBox-26nt
3xBBox-52nt
6xBBox-26nt
6xBBox-52nt
K-2
K-2/λ-4GIm

Figure S2

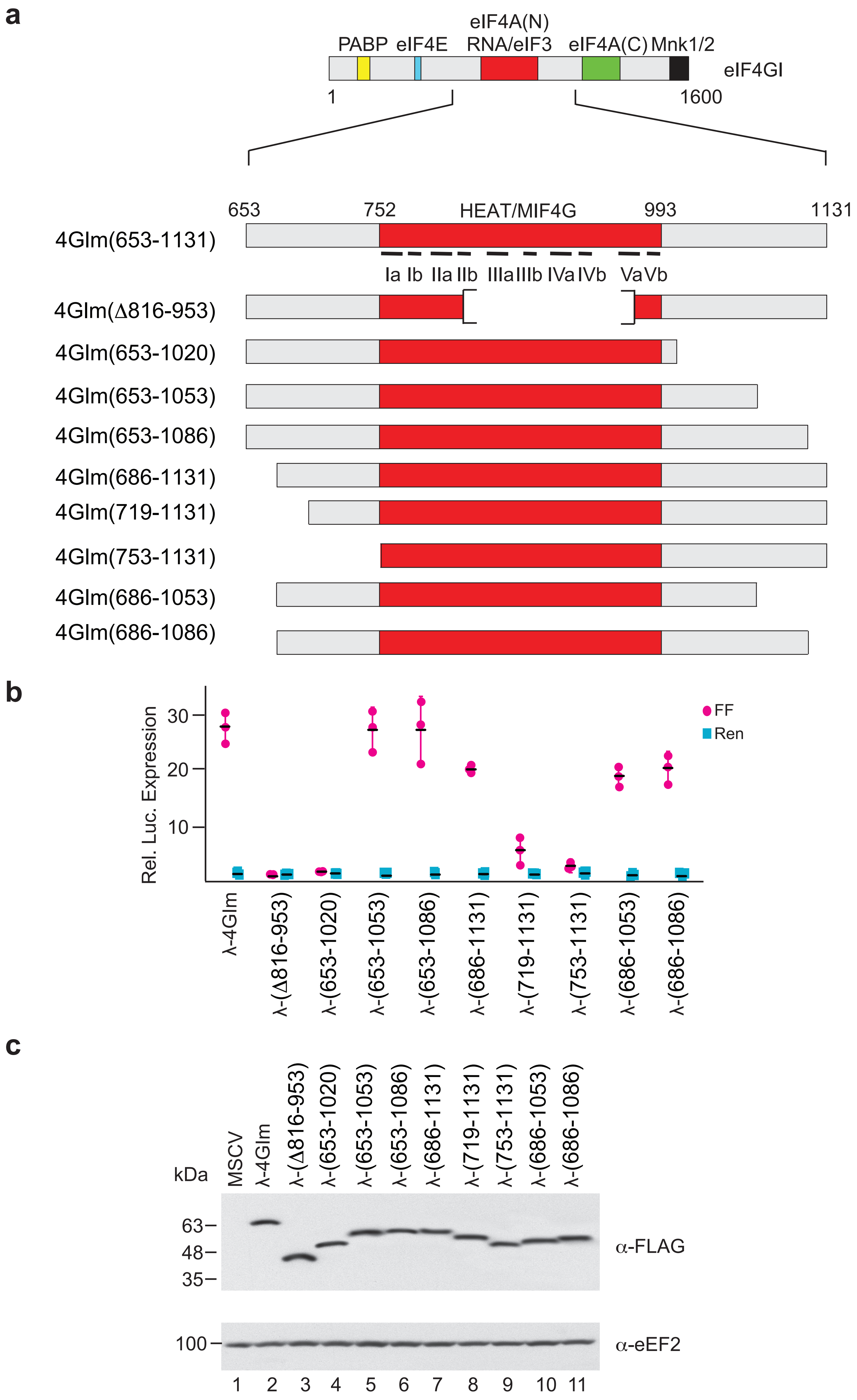
a
PABP
eIF4E
eIF4A(N)
RNA/eIF3
eIF4A(C)
Mnk1/2
eIF4GI
1
1600
653
752
HEAT/MIF4G
993
1131
4GIm(653-1131)
Ia Ib IIa IIb IIIa IIIb IVa IVb Va Vb
4GIm(Δ816-953)
4GIm(653-1020)
4GIm(653-1053)
4GIm(653-1086)
4GIm(686-1131)
4GIm(719-1131)
4GIm(753-1131)
4GIm(686-1053)
4GIm(686-1086)
b
Rel. Luc. Expression
30
20
10
FF
Ren
λ-4GIm
λ-(Δ816-953)
λ-(653-1020)
λ-(653-1053)
λ-(653-1086)
λ-(686-1131)
λ-(719-1131)
λ-(753-1131)
λ-(686-1053)
λ-(686-1086)
c
kDa
MSCV
λ-4GIm
λ-(Δ816-953)
λ-(653-1020)
λ-(653-1053)
λ-(653-1086)
λ-(686-1131)
λ-(719-1131)
λ-(753-1131)
λ-(686-1053)
λ-(686-1086)
63
48
35
α-FLAG
100
α-eEF2
1 2 3 4 5 6 7 8 9 10 11

Figure S3

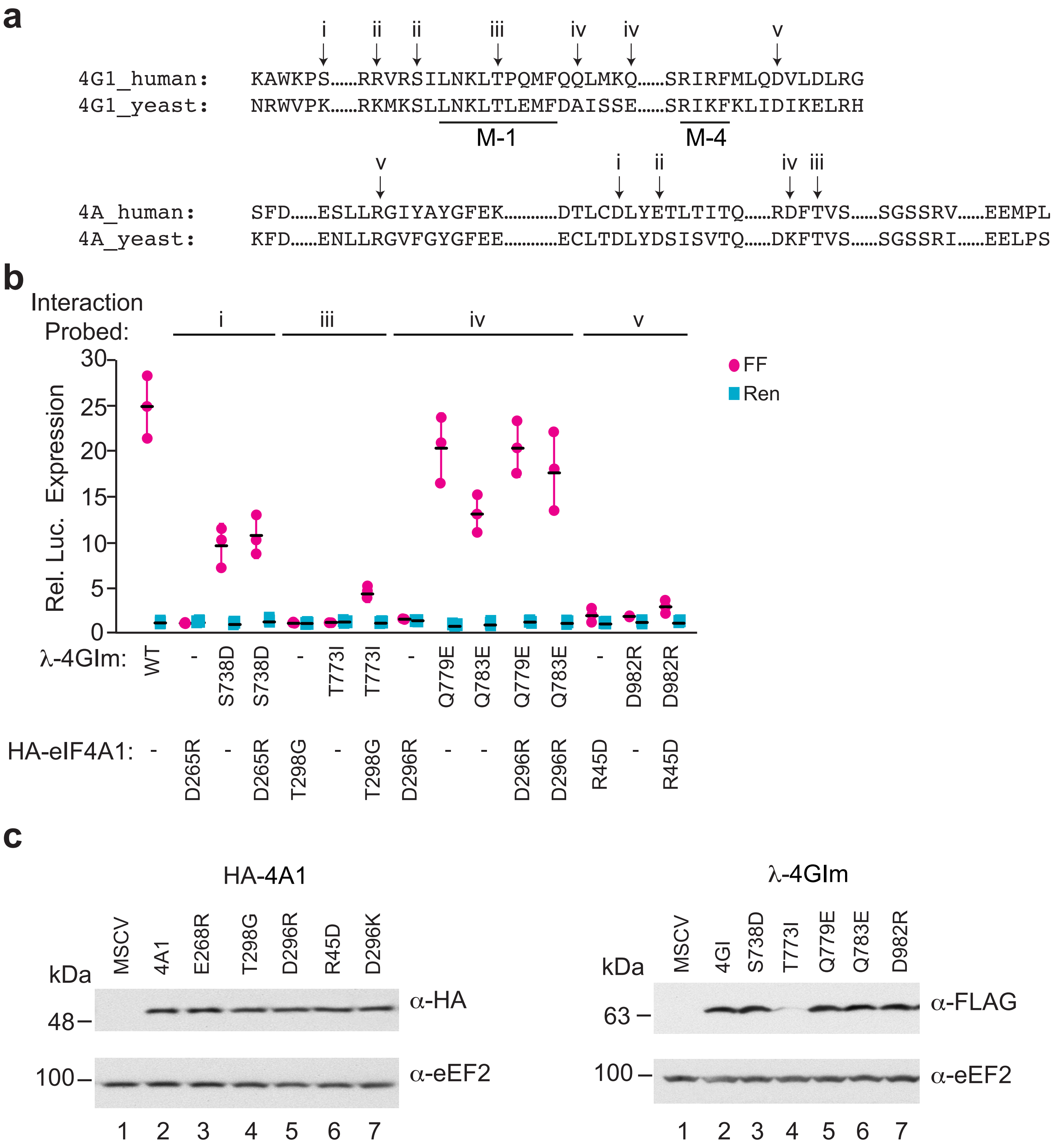
a
4G1_human: KAWKPS......RRVRSILNKLTPQMFQQLMKQ......SRIRFMLQDVLDLRG
4G1_yeast: NRWVPK......RKMKSLLNKLTLEMFDAISSE......SRIKFKLIDIKELRH
M-1
M-4
4A_human: SFD......ESLLRGIYAYGFEK............DTLCDLYETLTITQ......RDFTVS......SGSSRV......EEMPL
4A_yeast: KFD......ENLLRGVFGYGFEE............ECLTDLYDSISVTQ......DKFTVS......SGSSRI......EELPS
b
Interaction Probed:
Rel. Luc. Expression
FF
Ren
λ-4GIm:
HA-eIF4A1:
c
HA-4A1
kDa
MSCV
4A1
E268R
T298G
D296R
R45D
D296K
α-HA
α-eEF2
λ-4GIm
4GI
S738D
T773I
Q779E
Q783E
D982R
α-FLAG

Figure S4

a

```
4A1   1    MSASQDSRSRDNG-PDGMEPEGVIESNWNEIVDSFDDMNLSESLLRGIYAYGFEKPSAIQ   59
           MS      +R++G P+GM+P+GVIESNWNEIVD+FDDMNL ESLLRGIYAYGFEKPSAIQ
4A2   1    MSGGSADYNREHGGPEGMDPDGVIESNWNEIVDNFDDMNLKESLLRGIYAYGFEKPSAIQ   60

4A1   60   QRAILPCIKGYDVIAQAQSGTGKTATFAISILQQIELDLKATQALVLAPTRELAQQIQKV   119
           QRAI+PCIKGYDVIAQAQSGTGKTATFAISILQQ+E++ K TQALVLAPTRELAQQIQKV
4A2   61   QRAIIPCIKGYDVIAQAQSGTGKTATFAISILQQLEIEFKETQALVLAPTRELAQQIQKV   120

4A1   120  VMALGDYMGASCHACIGGTNVRAEVQKLQMEAPHIIVGTPGRVFDMLNRRYLSPKYIKMF   179
           ++ALGDYMGA+CHACIGGTNVR E+QKLQ EAPHI+VGTPGRVFDMLNRRYLSPK+IKMF
4A2   121  ILALGDYMGATCHACIGGTNVRNEMQKLQAEAPHIVVGTPGRVFDMLNRRYLSPKWIKMF   180

4A1   180  VLDEADEMLSRGFKDQIYDIFQKLNSNTQVVLLSATMPSDVLEVTKKFMRDPIRILVKKE   239
           VLDEADEMLSRGFKDQIY+IFQKLN++ QVVLLSATMP+DVLEVTKKFMRDPIRILVKKE
4A2   181  VLDEADEMLSRGFKDQIYEIFQKLNTSIQVVLLSATMPTDVLEVTKKFMRDPIRILVKKE   240

4A1   240  ELTLEGIRQFYINVEREEWKLDTLCDLYETLTITQAVIFINTRRKVDWLTEKMHARDFTV   299
           ELTLEGI+QFYINVEREEWKLDTLCDLYETLTITQAVIF+NTRRKVDWLTEKMHARDFTV
4A2   241  ELTLEGIKQFYINVEREEWKLDTLCDLYETLTITQAVIFLNTRRKVDWLTEKMHARDFTV   300

4A1   300  SAMHGDMDQKERDVIMREFRSGSSRVLITTDLLARGIDVQQVSLVINYDLPTNRENYIHR   359
           SA+HGDMDQKERDVIMREFRSGSSRVLITTDLLARGIDVQQVSLVINYDLPTNRENYIHR
4A2   301  SALHGDMDQKERDVIMREFRSGSSRVLITTDLLARGIDVQQVSLVINYDLPTNRENYIHR   360

4A1   360  IGRGGRFGRKGVAINMVTEEDKRTLRDIETFYNTSIEEMPLNVADLI   406
           IGRGGRFGRKGVAIN VTEEDKR LRDIETFYNT++EEMP+NVADLI
4A2   361  IGRGGRFGRKGVAINFVTEEDKRILRDIETFYNTTVEEMPMNVADLI   407
```

b

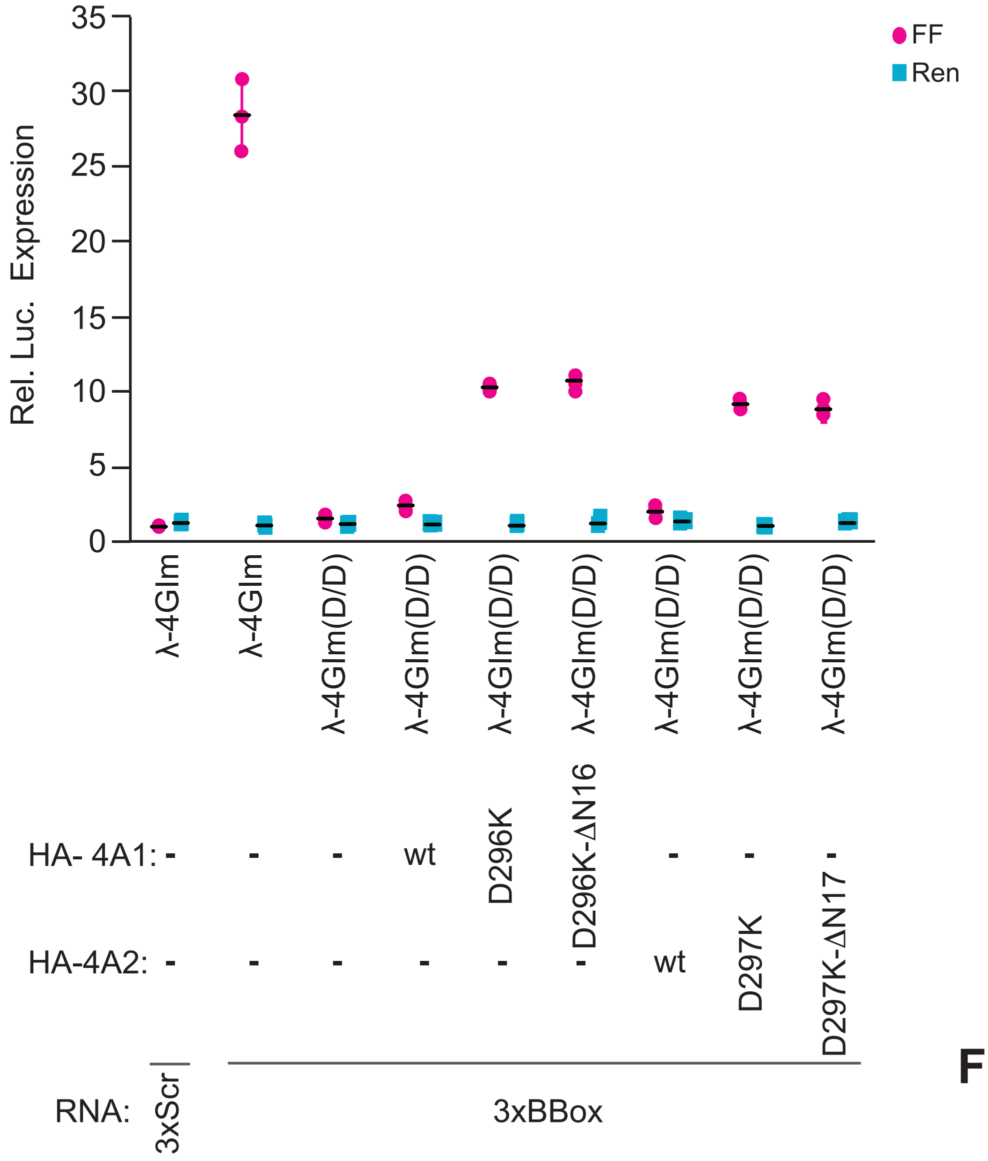

Figure S5

a

Motif Targeted	DEAD		HRIGRGGR		PTRELA
Mutation	DQAD	DEAH	R362K	R365K	PRRVAA
ATP Crosslinking [- RNA]	280%	760%	88%	200%	26%
ATP Crosslinking [+ RNA]	60%	410%	85%	74%	6%
ATPase	0%	340%	65%	62%	0%
RNA Binding	5%	123%	4%	7%	7%
Helicase Activity	0%	10%	0%	0%	0%

b

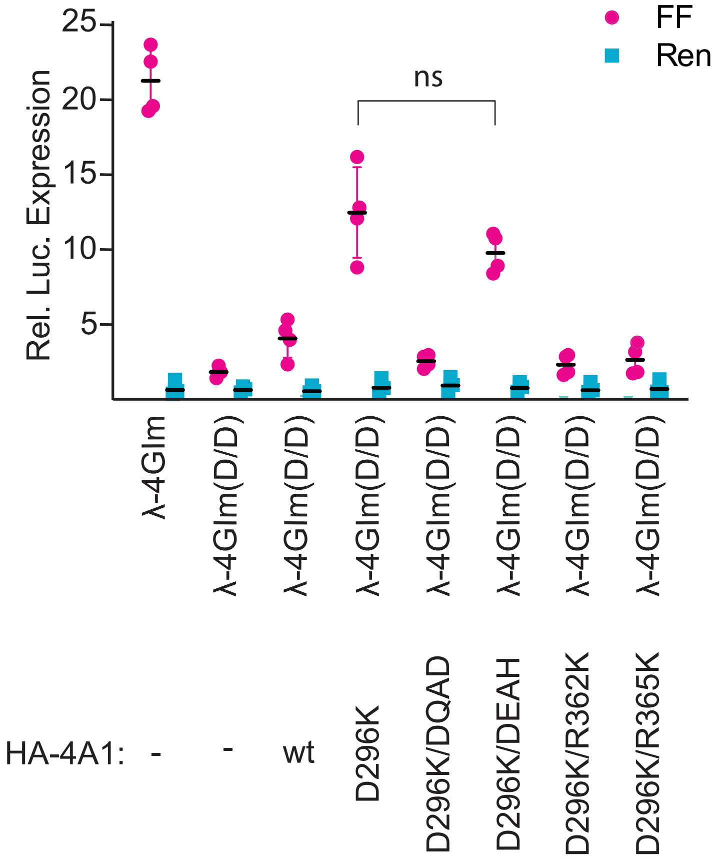

c

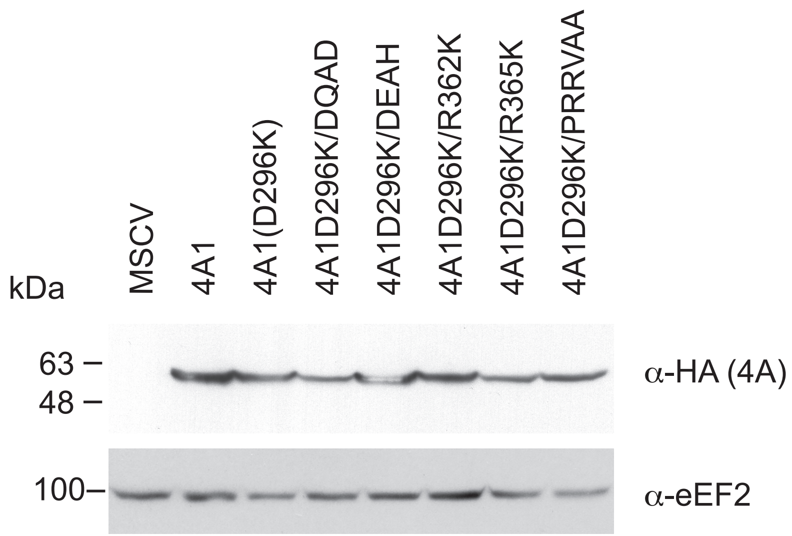

Figure S6

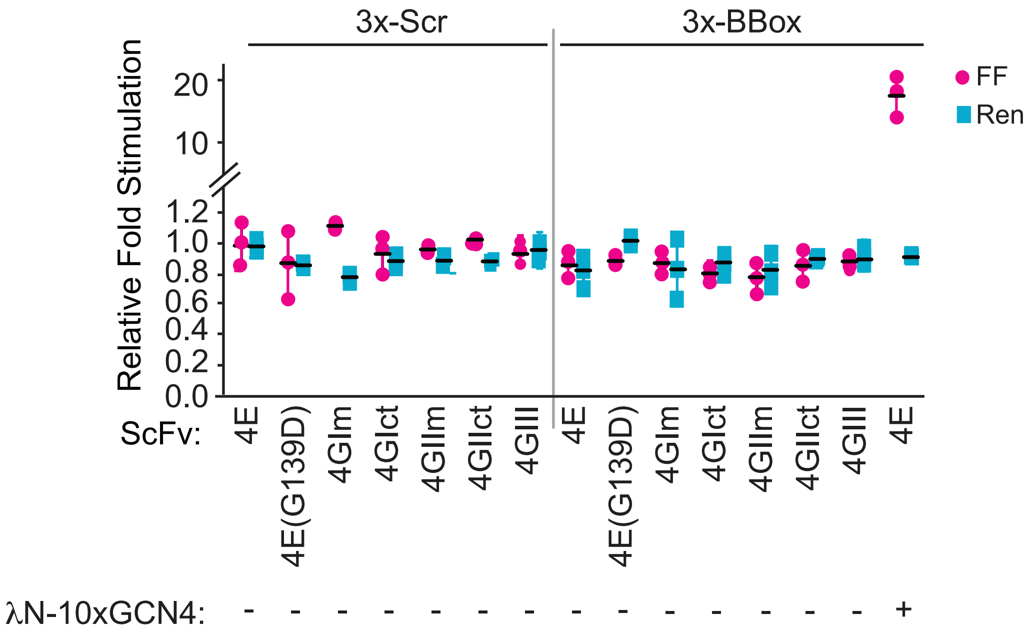
3x-Scr
3x-BBox
Relative Fold Stimulation
20
10
1.2
1.0
0.8
0.6
0.4
0.2
0.0
FF
Ren
ScFv:
4E
4E(G139D)
4GIm
4GIct
4GIIm
4GIIct
4GIII
4E
4E(G139D)
4GIm
4GIct
4GIIm
4GIIct
4GIII
4E
λN-10xGCN4:
- - - - - - - - - - - - - - +

Figure S7
